# An end-to-end machine-learning system for global eddy-resolving ocean forecast

**DOI:** 10.1126/sciadv.aeh9058

**Published:** 2026-11-13

**Authors:** Xiang Zhang, Yingzhe Cui, Furong Li, Shenghui Zhou, Guangnan Wu, Zhao Jing, Lixin Wu

**Affiliations:** ^1^ Department of Ocean Big Data and Artificial Intelligence, Laoshan Laboratory, Qingdao 266237, China.; ^2^ Frontiers Science Center for Deep Ocean Multispheres and Earth System and Key Laboratory of Physical Oceanography/Academy of Future Ocean, Ocean University of China, Qingdao 266100, China.; ^3^ Institute of Marine Science and Technology, Shandong University, Qingdao 266237, China.; ^4^ Qingdao Guoshi Technology Group Co. Ltd, Qingdao 266237, China.

## Abstract

Machine learning (ML) models, especially those based on deep neural networks, have achieved competitive forecast accuracy and greatly reduced computational burden compared to traditional numerical global ocean forecast systems (GOFSs). However, it remains difficult for these ML models to operate on their own, as they rely on the initial conditions generated by numerical GOFSs. In this study, we present WenHai-E2E, an end-to-end ML GOFS that replaces the entire pipeline of numerical GOFSs at deployment time and makes eddy-resolving global ocean forecast directly from multisource heterogeneous ocean observations, without requiring operational initial conditions from numerical GOFSs. WenHai-E2E outperforms a state-of-the-art numerical GOFS in the forecast accuracy. Our results demonstrate the promise of ML GOFSs to surpass numerical GOFSs and provide insight into the pathway for developing next generation of GOFSs.

## INTRODUCTION

The ocean is a chaotic system, where minor perturbations to initial conditions can amplify rapidly, significantly altering the trajectory of future ocean states (*1*–*3*). Consequently, operational ocean forecasting necessitates frequent observational corrections to mitigate forecast error accumulation. This is achieved through data assimilation (DA), which regularly integrates observations with prior forecasts to produce an optimal estimate of the current ocean state (the so-called analysis). This analysis then initializes a forecast model to produce estimates for the future ocean state. Together, the cycling between the forecast model and the DA model forms the core of global ocean forecast systems (GOFSs) that support a wide range of marine activities and management (*4*, *5*).

The forecast model is traditionally an ocean general circulation model (OGCM) that numerically discretizes and integrates the governing partial differential equations of the ocean forward in time on predefined grids. However, OGCMs suffer from discretization errors, imperfect parameterizations of unresolved processes, and uncertainties in the boundary conditions (*6*), degrading their fidelity of representing the complicated ocean dynamics and cross-sphere interactions. Furthermore, operating an OGCM could be computationally expensive and time-consuming, when its grid size becomes fine enough (less than ∼10 km) to resolve ocean mesoscale eddies having the majority of ocean kinetic energy (*7*, *8*). Machine learning (ML) models, especially those based on deep neural networks (DNNs), have become a promising substitute for OGCMs to make forecast. There is growing literature (*9*–*15*) demonstrating that ML forecast models can outperform their numerical counterparts (i.e., OGCMs) in forecast accuracy accompanied by orders of magnitude of increase in the computational efficiency.

Despite the advantages of ML forecast models over OGCMs, they cannot operate on their own. In particular, they rely on the initial conditions (i.e., the analysis) produced by the numerical GOFSs, in which case OGCMs still need to be run operationally (*9*–*15*). Moreover, the sparse in situ ocean observations and large ocean inertial make the short-term (daily to weekly timescales) forecast errors dominated by errors in the initial conditions, with the error growth during the forecast period being relatively minor (*16*, *17*). As the ML forecast models have to adopt the initial conditions from the numerical GOFSs, they inherit the initial condition errors from the numerical GOFSs so that their potential for further improving forecast accuracy is limited.

Recently, several pioneer studies have attempted to develop closed-loop ML forecast systems for the medium-range weather forecast (*18*–*21*). Aardvark Weather uses an ML encoder module mapping raw observations onto the variables required to initialize an ML forecast model (*18*). FengWu-Adas, FengWu-4DVar, and FuXi Weather assimilate observations with prior forecasts from an ML forecast model to produce the analysis (*19*–*21*), in a way similar as the forecast-assimilation cycling in the numerical weather prediction (NWP) systems. However, the quality of the initial conditions generated by these ML forecast systems still falls short of that produced by state-of-the-art NWP systems (*18*–*21*). Accordingly, at short forecast lead times, NWP systems still have a clear advantage over ML forecast systems in terms of the forecast accuracy (*18*–*21*). So far it remains not known whether ML forecast systems can surpass NWP systems. For GOFSs, related efforts have also been made. DeepDA reconstructs gridded ocean analyses from observations, but it does not include a forecast model (*22*). Therefore, whether ML GOFSs can surpass numerical GOFSs is still unclear.

In this study, we present WenHai-E2E, an end-to-end ML GOFS for short-term eddy-resolving forecast across the global upper ocean (0 to 2000 m). WenHai-E2E is designed to replace the conventional numerical GOFS pipeline at deployment time. Once trained, it makes forecast directly from multisource heterogeneous ocean observations, without relying on operational initial conditions from a numerical GOFS. We compare WenHai-E2E’s forecast performance with a state-of-the-art eddy-resolving numerical GOFS, i.e., the 1/12° operational ocean analysis and forecast system (*23*, *24*) (GLO12v4) from the France Mercator Océan (see the “GLO12v4” section in Materials and Methods). WenHai-E2E shows considerable improvement in the forecast accuracy compared to GLO12v4, demonstrating the promise of ML GOFSs to surpass numerical GOFSs.

## RESULTS

### Architecture of WenHai-E2E

WenHai-E2E is aimed to make global forecast of 1/12° daily averaged three-dimensional temperature, salinity and horizontal current in the upper 2000 m as well as the sea surface height (SSH) directly from multisource heterogeneous ocean observations. Although it is attractive to train a single monolithic DNN to achieve this goal, the training is probably challenging especially due to the sparsity of ocean observations (this is contrary to the weather forecast problem where observations are much denser). Instead, WenHai-E2E deals with this challenging problem via a modular Fusion-Forecast-Retrieval design (Fig. 1). Each module is feasible for training and physically interpretable.

**Fig. 1.**
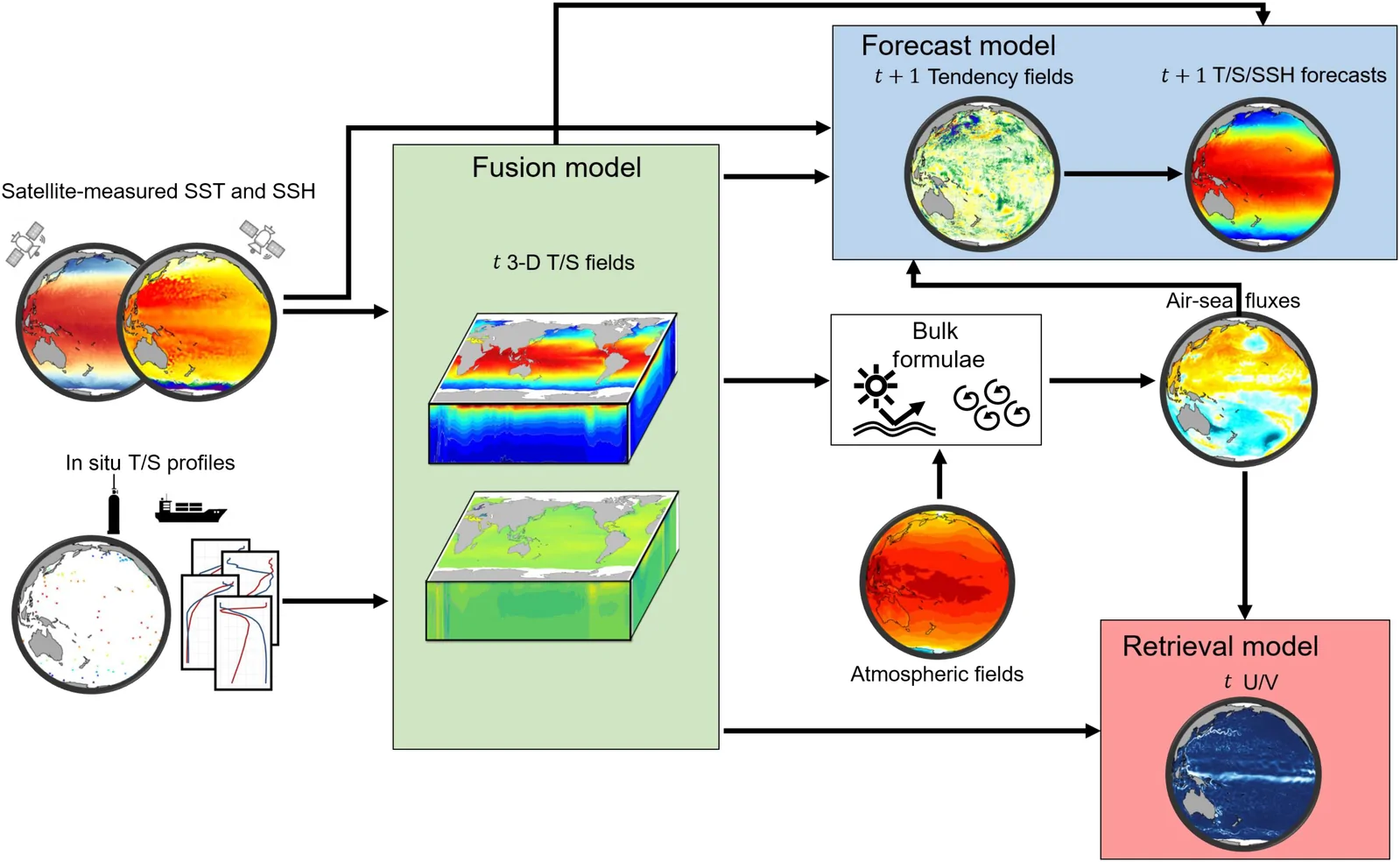
A schematic of WenHai-E2E’s architecture. Satellite-measured SST and SSH and in situ T/S profiles up to the current time t are first fused to construct the three-dimensional T/S fields (green block). Then, the three-dimensional T/S, SSH and surface atmosphere variables are input to a DNN outputting the temporal tendencies of three-dimensional T/S and SSH (blue block). The bulk formulae are incorporated into the DNN to transform surface atmosphere and ocean variables to air-sea fluxes. The tendencies are added back to the three-dimensional T/S and SSH on the day t to yield their forecast values on the day t+1. Last, another DNN is trained to retrieve the three-dimensional horizontal currents based on the air-sea fluxes, SSH and three-dimensional T/S at the same time (red block). The above processes can be iterated to generate a sequence of forecasts.

The fusion model merges gridded satellite-measured sea surface temperature (SST) and SSH and in situ temperature/salinity (T/S) profiles (see the “Observational datasets” section in Materials and Methods) via linear ML methods to construct three-dimensional gridded T/S fields as the initial condition of the forecast (see the “Fusion model” section in Materials and Methods). We do not use the satellite-measured sea surface salinity, as the gridded product is released with a 1-week delay so that it is not available for the forecast purpose (*25*). It should be noted that the in situ T/S profiles are sparse, being typically one profile within a 300 km–by–300 km area every ten days on average (*26*). To improve the construction accuracy of three-dimensional T/S, in situ T/S profiles collected before the time in question are used in view of the long memory of the ocean. The rationality of using linear ML methods has been verified by Zhang *et al.* (*27*) who exploit the quasi-geostrophic dynamics (*28*) and demonstrate that the subsurface hydrographic variations depend linearly on the variations of satellite-measured quantities. Furthermore, the linear ML methods are more resistant to the overfitting arising from sparse observations compared to complicated nonlinear models such as DNNs. The constructed three-dimensional T/S fields are analogous to the analysis fields generated by the DA model in the numerical GOFSs. However, they are obtained solely from the observations without incorporating prior forecasts. This avoids the accumulation of prior forecast errors in the initial condition. In particular, in view of the forecast model is an ML model lacking of complete physical constraints, decoupling the initial condition from the prior forecasts will be useful for ML GOFSs to operate steadily without gradual amplification of physically unrealistic features.

The forecast model propagates SSH and three-dimensional T/S forward in time under atmospheric forcing in an autoregressive way (see the “Forecast model” section in Materials and Methods). Specifically, it has a Swin-Transformer backbone (*29*), takes as input the SSH, three-dimensional T/S and surface atmosphere variables on some day and outputs the temporal tendency of SSH and three-dimensional T/S. As both the input and output of the forecast model are gridded datasets, it can be trained efficiently based on the historical reanalysis data (see the “GLORYS reanalysis” section in Materials and Methods). To explicitly represent the atmospheric forcings, the bulk formulae (*30*) are incorporated into the forecast model, transforming the surface atmosphere variables into the air-sea momentum, heat, and freshwater fluxes. It should be noted that the surface atmospheric variables are obtained from the same weather forecast product used by GLO12v4 (see the “Atmospheric dataset” section in Materials and Methods), which is necessary for a fair comparison between WenHai-E2E and GLO12v4.

The retrieval model diagnoses the three-dimensional horizontal currents based on the air-sea fluxes, SSH, and three-dimensional T/S (see the “Retrieval model” section in Materials and Methods). It shares similar neural network configurations as the forecast model. Again, it can be trained efficiently based on the historical reanalysis data, as both the model input and output are gridded datasets. In principle, primarily the horizontal currents of balanced motions (*3*, *28*) such as geostrophic flows and Ekman flows can be diagnosed. The unbalanced horizontal currents are underdetermined and thus neglected. This neglection reflects the limitation of ocean observations used for operational oceanography that fail to provide sufficient information on unbalanced motions.

### Forecast performance of WenHai-E2E

The forecast performance of WenHai-E2E and GLO12v4 is evaluated following the GODAE Ocean-View Intercomparison and Validation Task Team (IV-TT) Class 4 framework (*16*). The ocean observations involved in the evaluation include the SST and 15-m horizontal current measured by drifting buoys, T/S profiles measured by Argo profiling floats and level 3 along-track sea level anomaly (SLA) measured by satellite altimeters (see the “Observational datasets” section in Materials and Methods). The root mean square error (RMSE; see the “Verification metrics” section in Materials and Methods) is used to quantify the forecast performance.

WenHai-E2E achieves lower RMSE for all the ocean variables throughout the 10-day forecast period, compared to GLO12v4. When forecasting 10 days in advance, WenHai-E2E has an RMSE for SST, SLA, 15-m zonal current, and 15-m meridional current 25.0, 49.6, 20.2, and 25.4% lower than GLO12v4 (Fig. 2). As to the 10-day-led forecast of temperature profile, the RMSE of WenHai-E2E is lower than that of GLO12v4 throughout the upper 2000 m (fig. S1A). So is the case for the salinity profile forecast (fig. S1B). The vertical mean RMSE for the temperature (salinity) profile forecast by WenHai-E2E is 34.5% (26.9%) lower than that by GLO12v4 (Fig. 2). The forecast performances of WenHai-E2E and GLO12v4 vary spatially and seasonally (figs. S2 and S3). Nevertheless, the RMSEs of WenHai-E2E for all the ocean variables are consistently lower than their counterparts of GLO12v4 regardless of the space and season.

**Fig. 2.**
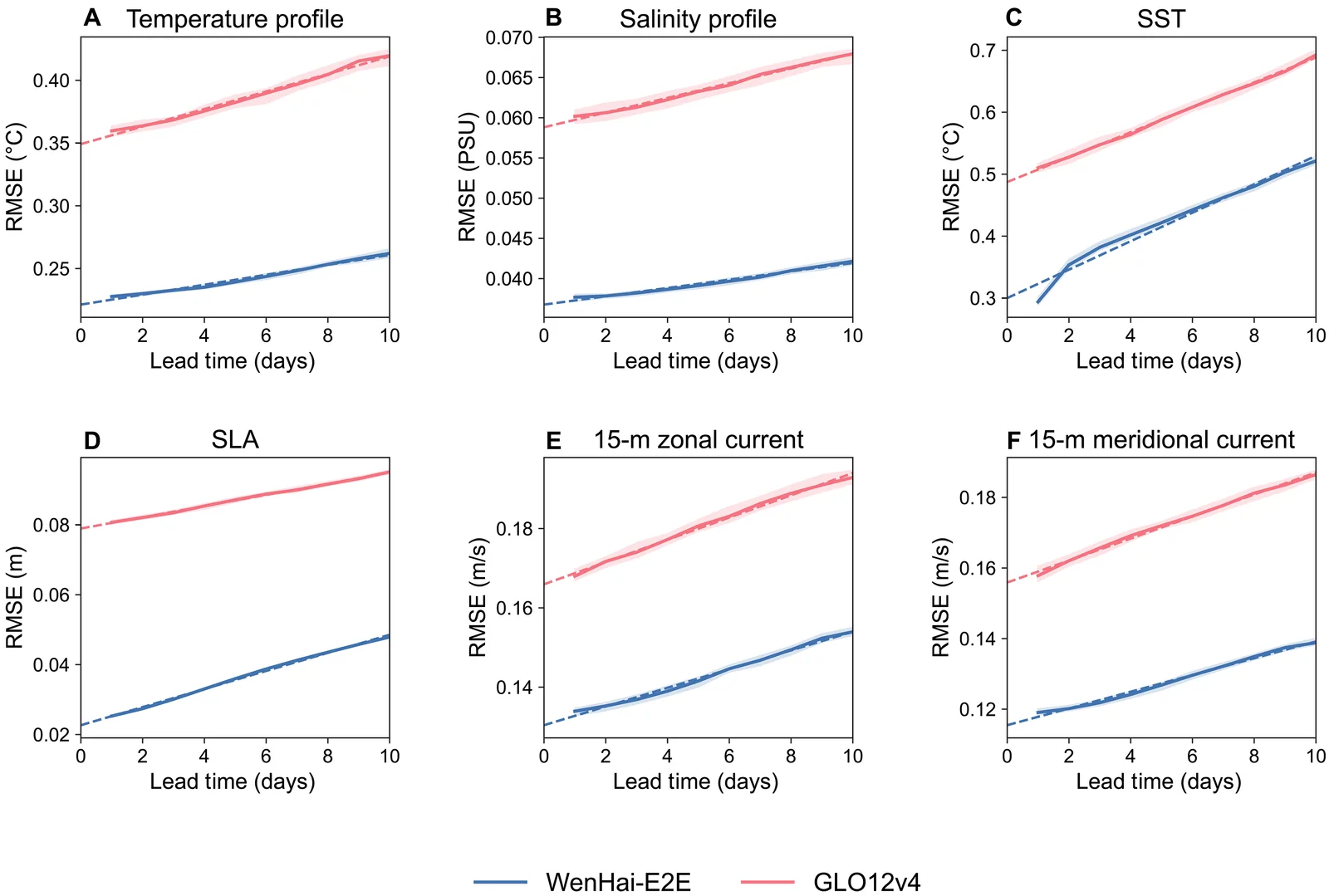
RMSE of WenHai-E2E (blue) and GLO12v4 (red) at different forecast lead times. Forecast RMSE (the lower, the better) of the temperature profile (**A**), salinity profile (**B**), SST (**C**), SLA (**D**), 15-m zonal current (**E**), and 15-m meridional current (**F**) averaged globally as a function of forecast lead time. The zero-lead time represents the initial conditions whose RMSE is computed based on the linear fit (dashed line) of forecast RMSE. The RMSE for the temperature and salinity profile forecast is vertically averaged over the upper 2000 m. The shading corresponds to the 90% confidence interval estimated from a bootstrap method.

The outperformance of WenHai-E2E over GLO12v4 could be attributed to superiority of the forecast model and/or initial condition of WenHai-E2E. We first evaluate the performance of the forecast model quantified by the persistence skill score (PSS) (see the “Verification metrics” section in Materials and Methods). A positive (negative) PSS indicates superiority (inferiority) over the persistent forecast assuming that the future ocean state were the same as the present. GLO12v4 can outperform the persistent forecast only for SST and 15-m horizontal currents, which is a common problem for eddy-resolving numerical GOFSs (Fig. 3) (*16*). In contrast, WenHai-E2E beats the persistent forecast for all the ocean variables (fig. S4). In particular, there is a positive trend in the PSS of WenHai-E2E, suggesting that advantage of WenHai-E2E over the persistent forecast becomes more evident with the increasing forecast lead time (Fig. 3). The PSS of GLO12v4 shows a positive trend only for the SST. Nevertheless, its slope is lower than that of WenHai-E2E. In summary, the forecast model of WenHai-E2E has good skills in predicting the ocean evolution and outperforms that of GLO12v4.

**Fig. 3.**
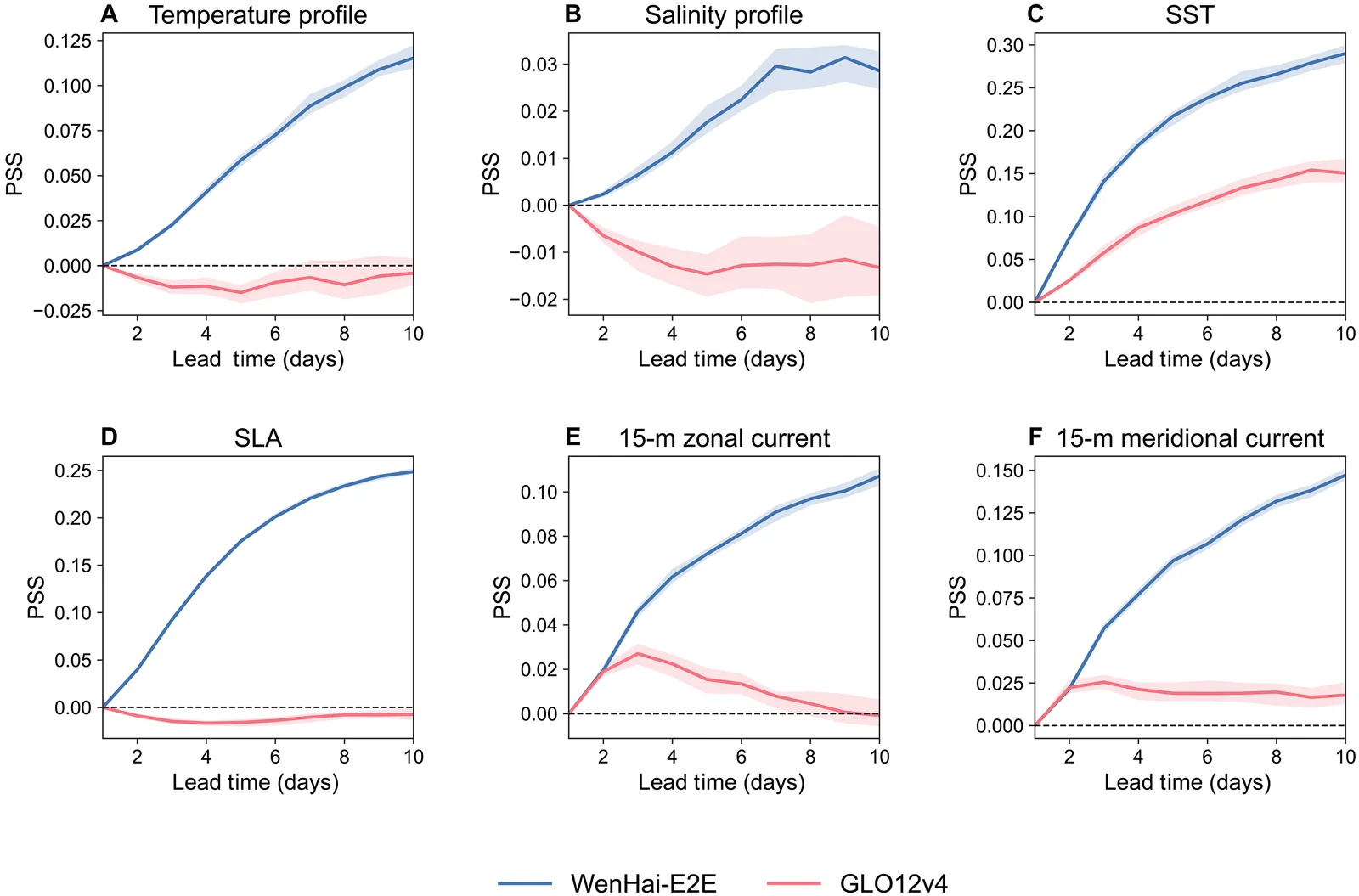
PSS of WenHai-E2E (blue) and GLO12v4 (red) at different forecast lead times. PSS (the higher, the better) of the temperature profile (**A**), salinity profile (**B**), SST (**C**), SLA (**D**), 15-m zonal current (**E**), and 15-m meridional current (**F**) averaged globally as a function of forecast lead time. For the temperature and salinity profile forecast, the PSS is computed based on the vertically averaged RMSE over the upper 2000 m. The shading corresponds to the 90% confidence interval estimated from a bootstrap method.

We next evaluate the quality of initial conditions of WenHai-E2E and GLO12v4. As the SST, SLA, and T/S observations used in the GODAE Ocean-View IV-TT Class 4 are used by the fusion model of WenHai-E2E and assimilation model of GLO12v4, the RMSEs of initial conditions based on these observations are essentially the in-sample errors (*31*) and thus do not provide reliable measurement of the quality of initial conditions. For this sake, the quality of initial conditions of WenHai-E2E and GLO12v4 is validated against temperature profiles from Autonomous Pinniped Bathythermograph (APB) and 15-m horizontal currents from drifting buoys that are not assimilated/fused by the two forecast systems (see the “Observational datasets” section in Materials and Methods). The APB temperature profiles are confined to the Southern Ocean with strongly uneven spatial distribution, whereas the drifter-measured 15-m horizontal currents spread across the global ocean and are more evenly distributed (fig. S5).

The RMSEs of temperature profiles of WenHai-E2E and GLO12v4 initial conditions share similar vertical structures, both peaking in the thermocline where the temperature is most variable (Fig. 4A). However, the RMSE of WenHai-E2E is evidently smaller than that of GLO12v4, reducing by 6.4 to 20.3% depending on the depth. As to the 15-m horizontal currents, the RMSE for zonal (meridional) current is 0.17 m/s (0.15 m/s) for the GLO12v4’s initial condition but is reduced to 0.13 m/s (0.12 m/s) for the WenHai-E2E’s initial condition (Fig. 4B). Although these limited independent test datasets prohibit a comprehensive evaluation, they lend supports to the superiority of WenHai-E2E over GLO12v4 in its initial condition quality.

**Fig. 4.**
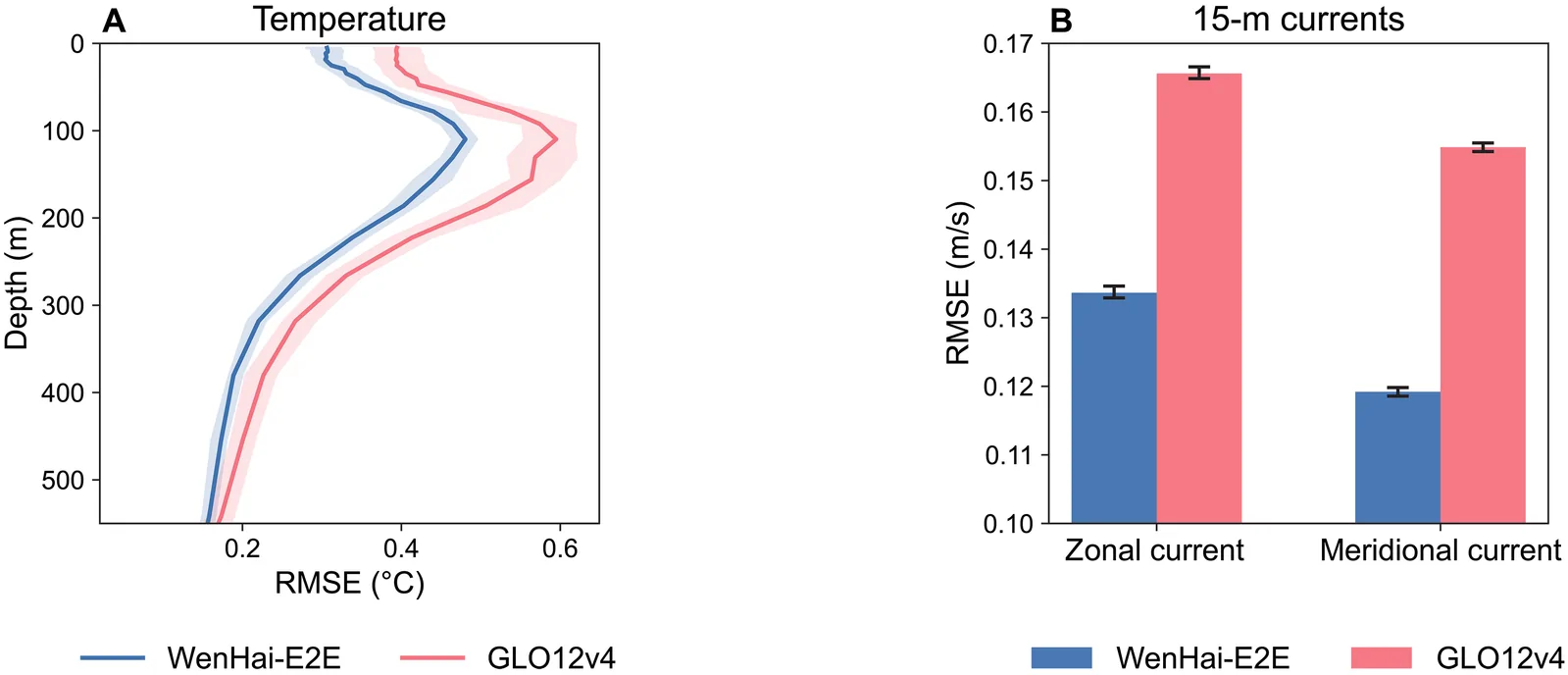
RMSE of initial conditions of WenHai-E2E (blue) and GLO12v4 (red). (**A**) RMSE of initial condition of temperature profile evaluated based on the APB as a function of depth. (**B**) RMSE of initial conditions of 15-m zonal and meridional currents evaluated based on the drifting buoys. The shading in (A) and error bar in (B) correspond to the 90% confidence interval estimated from a bootstrap method.

An alternative way to estimate the RMSE of initial conditions is to extrapolate the forecast RMSE to the initial time (corresponding to the 0-day forecast lead) by exploiting the fact that the forecast RMSEs for both WenHai-E2E and GLO12v4 are approximately linear functions of the forecast lead time. The RMSE of initial condition of WenHai-E2E is evidently reduced for all the ocean variables, compared to GLO12v4 (Fig. 2). In particular, the reduction of RMSE of initial condition makes a major contribution to the reduced forecast RMSE in WenHai-E2E. Therefore, despite the superiority of the WenHai-E2E’s forecast model over the GLO12v4’s counterpart, it is the higher quality of initial condition of WenHai-E2E that accounts primarily for the smaller forecast RMSE.

The RMSE is a conventional point-to-point verification metric adopted by the GODAE Ocean-View IV-TT Class 4 framework. However, it has been recognized that the blurring effect of ML forecast could reduce the RMSE due to the double penalty issue (*32*–*34*), making the ML GOFSs verify superficially better than numerical GOFSs via lowering their effective resolution. To overcome the limitation of RMSE, a neighborhood-based verification metric, i.e., the continuous ranked probability score (CRPS; see the “Verification metrics” section in Materials and Methods) (*35*), is adopted to compare the performance of WenHai-E2E and GLO12v4. The CRPS has been routinely applied to evaluating high-resolution oceanic and atmospheric forecast systems (*36*, *37*). In particular, lowering the effective resolution of the forecasts, albeit potentially capable of reducing the RMSE, leads to increase of the CRPS (*12*, *36*, *38*).

Evaluation of WenHai-E2E and GLO12v4 based on the CRPS yields the same conclusion as evaluation based on the RMSE, that is, the CRPS of WenHai-E2E is lower than that of GLO12v4 for all the ocean variables across all the forecast lead times (Fig. 5). This consistency suggests that the lower forecast RMSE of WenHai-E2E than GLO12v4 is not an artifact caused by the blurring effect of WenHai-E2E but genuinely reflects its better capability in forecasting the ocean state. Specifically, for the forecasts led by 10 days, the CRPS of WenHai-E2E is 20.3% lower than that of GLO12v4 for the temperature profile, 13.8% lower for the salinity profile, 13.8% lower for the SST, 57.3% lower for the SLA, and 8.8% (12.9%) lower for the 15-m zonal (meridional) current, respectively. However, unlike the forecast RMSE, both the higher-quality initial condition and superiority of the forecast model of WenHai-E2E contribute importantly to the reduced forecast CRPS (Fig. 5).

**Fig. 5.**
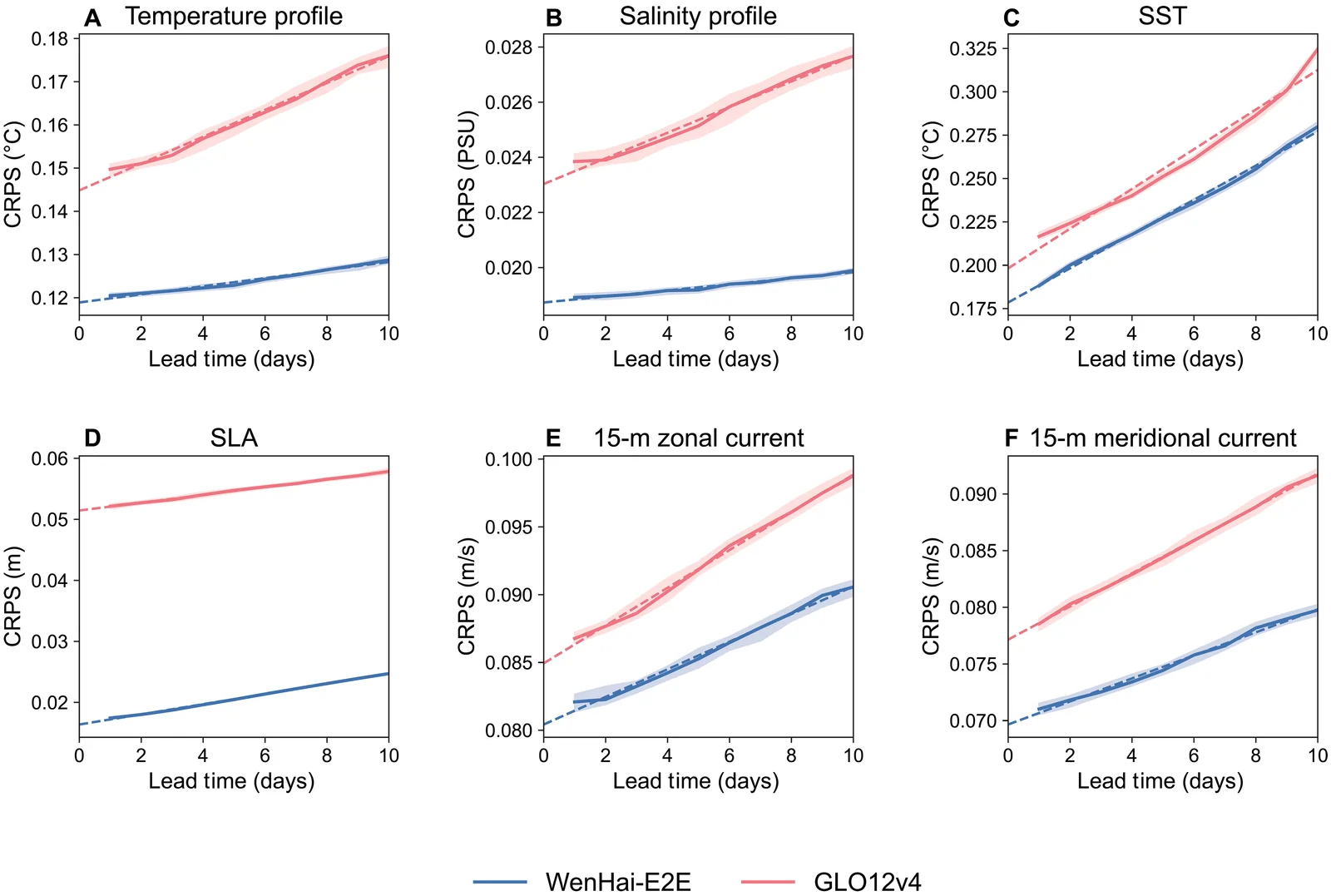
CRPS of WenHai-E2E (blue) and GLO12v4 (red) at different forecast lead times. Forecast CRPS (the lower, the better) of the temperature profile (**A**), salinity profile (**B**), SST (**C**), SLA (**D**), 15-m zonal current (**E**), and 15-m meridional current (**F**) averaged globally as a function of forecast lead time. The zero-lead time represents the initial conditions whose CRPS is computed based on the linear fit (dashed line) of forecast CRPS. The CRPS for the temperature and salinity profile forecast is vertically averaged over the upper 2000 m. The shading corresponds to the 90% confidence interval estimated from a bootstrap method.

### Capability of WenHai-E2E in forecasting mesoscale eddies

As WenHai-E2E is designed as an eddy-resolving forecast system, it is necessary to evaluate its capacity of forecasting the evolution of mesoscale eddies. This is done first based on an illustrative case study of a mesoscale eddy merging event, followed by a comprehensive assessment on the performance of WenHai-E2E in forecasting mesoscale eddies globally, including their location, intensity and radius.

A mesoscale eddy-merging event occurred in the western North Atlantic (36°N to 41°N, 58°W to 63°W) during 20 to 30 March 2025. Satellite observations on 20 March 2025 revealed two cyclonic eddies with their negative SLA centered at 61.5°W, 38.7°N and 59.1°W, 38°N on 20 March, respectively (Fig. 6A). Over the subsequent 10 days, the two eddies exhibited a steady migration toward each other and merged into an intensified cyclonic eddy with its radius reaching approximately twice as the individual eddies (Fig. 6B). Eddy merging involves highly nonlinear dynamics (*39*, *40*) and is typically challenging for numerical GOFSs to forecast. The GLO12v4 fails to forecast this eddy merging event (Fig. 6D), whereas WenHai-E2E captures this phenomenon qualitatively well (Fig. 6C).

**Fig. 6.**
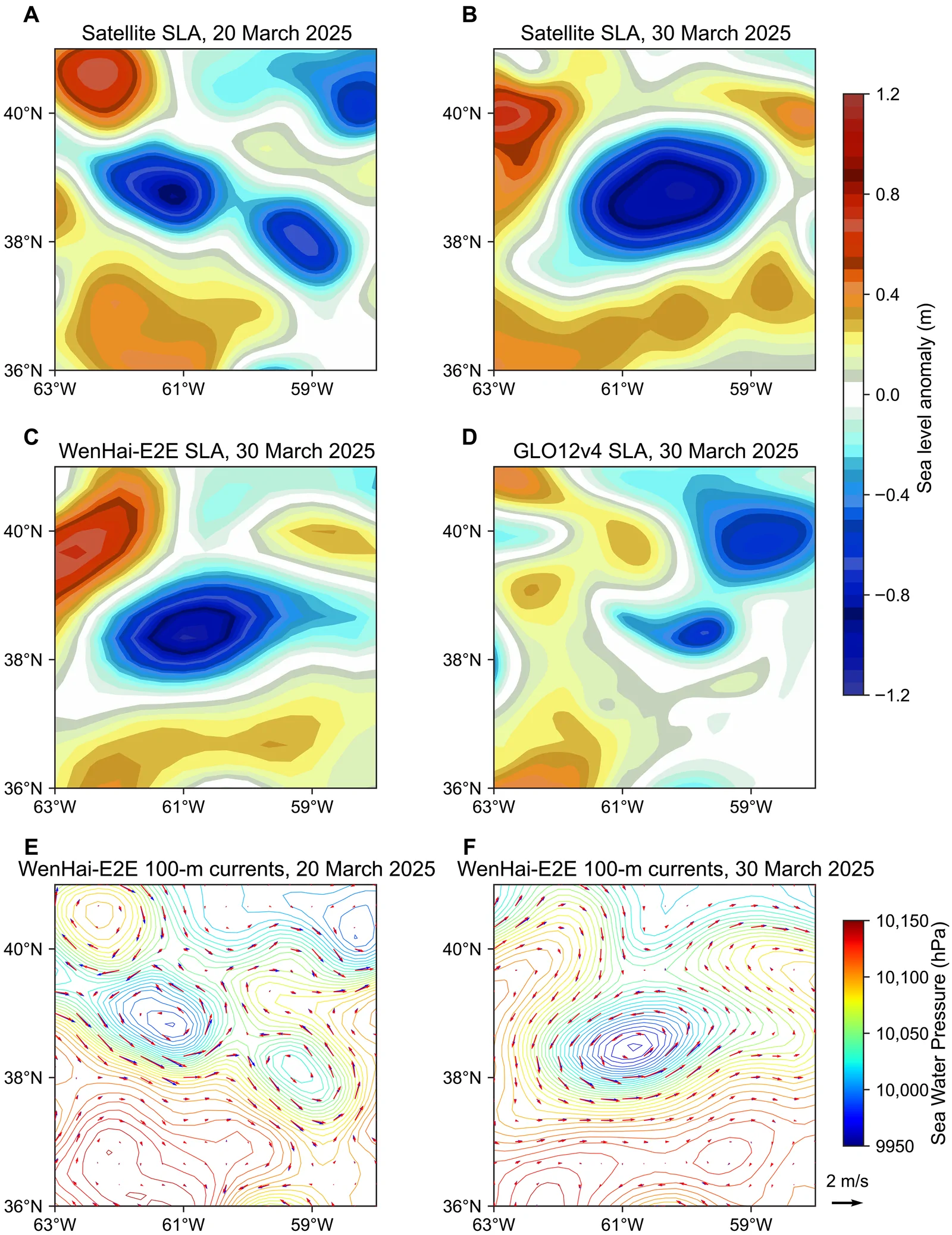
Illustration of physical consistency of WenHai-E2E’s forecast based on a mesoscale eddy merging event in the western North Atlantic. (**A**) Satellite-measured SLA on 20 March 2025 when the forecast is initialized. (**B** to **D**) SLA on 30 March 2025 from satellite observations and WenHai-E2E’s and GLO12v4’s 10-day-led forecasts, respectively. (**E** and **F**) Horizontal currents (blue) and geostrophic currents (red) at 100 m on 20 March 2025 derived from WenHai-E2E’s initial condition (E) and on 30 March 2025 derived from WenHai-E2E’s 10-day-led forecast. Contours in (E) and (F) denote the sea water pressure at 100 m with an interval of 5 hPa.

Mesoscale eddies, including the ones shown in Fig. 6, have a Rossby number (Ro) much smaller than unity (*3*, *41*). In the absence of frictional and topographic effects, a leading-order balance for flows with a small Ro is the geostrophic balance, where the horizontal pressure gradient is balanced by the Coriolis force (*3*, *28*). Accordingly, the horizontal current should vary substantially during the eddy merging to adjust to the substantial change of SLA, providing a testbed to assess whether WenHai-E2E is able to capture this leading-order balance for mesoscale eddies. Figure 6 (E and F) compares the horizontal currents on 20 and 30 March at 100 m where the frictional effect becomes negligible (the Ekman layer depth estimated from the surface wind stress is around 60 m). The horizontal currents between these two days differ substantially from each other, as expected from the rapid evolution of horizontal currents during the eddy merging. Despite evolving rapidly, the horizontal currents remain close to the geostrophic currents (see the “Computation of geostrophic currents” section in Materials and Methods). The closeness between the horizontal current forecast by WenHai-E2E and the geostrophic component is not limited to this particular event but holds across the global ocean wherever the Ro is small (fig. S6). This lends supports that WenHai-E2E’s forecast does not violate the leading-order balance for mesoscale eddies.

We next conduct a comprehensive assessment on the performance of WenHai-E2E in forecasting mesoscale eddies globally. Mesoscale eddies and their properties are identified from the SLA fields using an eddy-identification algorithm (see the “Eddy identification” section in Materials and Methods). Here, the mesoscale eddies identified from the gridded SLA from satellite observations are treated as the ground truth for validation. It should be noted that the gridded SLA is constructed by merging along-track SLA from multiple altimeters and subjected to uncertainties in identifying mesoscale eddies with relatively small radius and amplitude. For this reason, the assessment is confined to mesoscale eddies with radius and amplitude larger than 50 km and 5 cm, respectively. Despite the removal of small or weak mesoscale eddies, the following comparison should be treated as qualitative rather than quantitative, in view of the uncertainties of gridded SLA.

WenHai-E2E consistently outperforms GLO12v4 in forecasting occurrence of mesoscale eddies and their properties throughout the forecast lead times (Fig. 7; see the “Eddy identification” section in Materials and Methods). For the forecasts led by 10 days, the hit rate, miss rate, and false alarm rates of WenHai-E2E are 69.1, 30.9, and 24.4% compared to 40.6, 59.5, and 55.5% of GLO12v4 (Fig. 7, A to C). In terms of mesoscale eddies whose occurrence is successfully forecast by both WenHai-E2E and GLO12v4, WenHai-E2E has better accuracy in forecasting the eddy properties. The 10-day-led RMSEs of eddy center position, eddy radius, and eddy amplitude forecast by WenHai-E2E are 50.0, 32.0, and 30.3% lower than their counterparts of GLO12v4, respectively (Fig. 7, D to F).

**Fig. 7.**
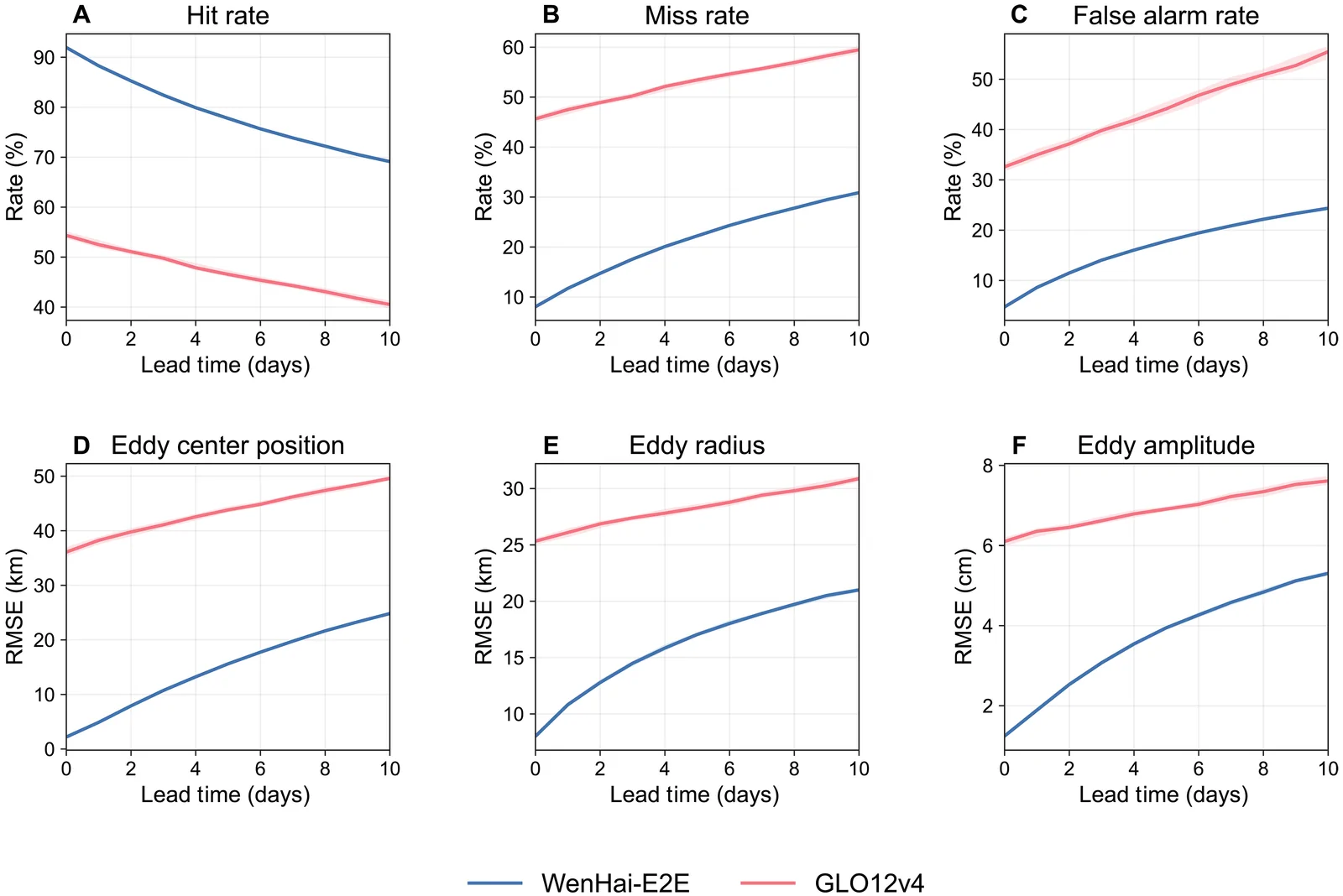
Performance of WenHai-E2E (blue) and GLO12v4 (red) at different forecast lead times. Hit rate (the higher, the better) (**A**), miss rate (the lower, the better) (**B**), and false alarm rate (the lower, the better) (**C**) of the forecast, and RMSE (the lower, the better) of forecast eddy center position (**D**), eddy radius (**E**), and eddy amplitude (**F**) as a function of forecast lead time. The shading corresponds to the 90% confidence interval estimated from a bootstrap method.

## DISCUSSION

In this study, we present WenHai-E2E, an end-to-end ML GOFS that directly produces eddy-resolving ocean forecasts globally from multisource heterogeneous observations, without requiring operational initial conditions from a numerical GOFS at deployment time. It operates through three ML models: a fusion model that constructs three-dimensional T/S fields from satellite-measured SST and SSH and in situ T/S profiles and provides the initial condition for forecast; a forecast model that autoregressively makes forecast for SSH and three-dimensional T/S; and a retrieval model that derives three-dimensional horizontal currents. WenHai-E2E outperforms the state-of-the-art eddy-resolving numerical GOFS (i.e., GLO12v4) for forecasting T/S profile, SST, SLA, and near-surface horizontal currents led by 1 day to at least 10 days when forced by the same atmospheric forecast product. The outperformance of WenHai-E2E is attributed both to the higher quality of initial condition and to the superiority of the forecast model. In particular, unlike numerical GOFSs, the initial condition of WenHai-E2E is decoupled from the forecast model so that it avoids the accumulation of prior forecast errors in the initial condition. This guarantees that WenHai-E2E can operate from a consistently high-quality starting point for each forecast cycle.

There are some limitations in WenHai-E2E despite its good performance. First, as a data-driven GOFS, WenHai-E2E’s performance is fundamentally tied to the quality and coverage of its training data from historical observations and reanalysis. Any systematic biases in the data, particularly in regions that do not have sufficient high-quality observations, are likely embedded into the ML models and degrade their performance. Second, WenHai-E2E tends to generate smoother forecast than numerical GOFSs, as implied by the reduced superiority of WenHai-E2E over GLO12v4 in terms of CRPS than RMSE. This is largely because that the current satellite and in situ observations used by operational ocean forecasting are unable to resolve small-scale ocean motions so that the initial condition constructed by the fusion model does not contain these variabilities. Third, although WenHai-E2E shows promising physical consistency, it lacks hard constraints enforcing fundamental conservation laws of mass, momentum, and energy. This is acceptable for short-term forecast but may cause problems for prediction at seasonal and longer time scales. Fourth, WenHai-E2E is only capable of forecasting the daily mean ocean state, neglecting the diurnal cycle and internal waves in the ocean. Last, WenHai-E2E does not address the sea ice, limiting its usability in sea ice–covered areas. We believe that future directions to improve the accuracy and resolution of ML GOFSs include using higher-quality training data, exploiting the newly developed observation platforms such as the Surface Water and Ocean Topography Mission (*42*, *43*), implementing explicit physical consistency modules in ML models (*44*, *45*), and incorporating the ML models for high-frequency ocean processes and sea ice (*46*, *47*).

Our results demonstrate the promise of ML GOFSs to operate on their own at deployment time and surpass numerical GOFSs, providing insight into developing new generation of GOFSs. However, it is important to acknowledge that the development and performance of WenHai-E2E are underpinned by the high-quality reanalysis datasets generated by numerical GOFSs. These reanalysis datasets provide essential training targets that enable ML models to learn the complicated ocean dynamics. Therefore, developing ML GOFSs will continue to rely on numerical GOFSs during model training. Further progress of ocean forecasts will require in-depth integration of advances in ML and the operational oceanography.

## MATERIALS AND METHODS

### GLO12v4

GLO12v4 is the latest version of the Operational Mercator global ocean analysis and forecast system, which delivers daily updated 10-day global ocean forecasts. It comprises an ocean component (NEMO3.6) that has a 1/12° horizontal resolution and 50 vertical levels, coupled with a multicategory sea ice component (LIM3) (*48*). Satellite-measured SLA, SST and sea ice concentration, and in situ T/S profiles are assimilated using a reduced-order Kalman filter.

The GLO12v4 forecast is initialized from hindcast simulation of GLO12v4 spanning the last 24 hours leading to real time (i.e., −24 to 0 hours). The atmospheric forecast product during the forecast period is provided by the European Centre for Medium-Range Weather Forecasts (ECMWF) Integrated Forecasting System (IFS) HRES forecast product (*49*). To evaluate forecast performance, we collect GLO12v4 forecasts along with their initial conditions and atmospheric forecast product during April 2024 to March 2025.

### Observational datasets

We integrate satellite-measured SST and SSH with in situ T/S profiles to construct three-dimensional temperature and salinity fields as the initial conditions for ocean forecast. The SST data are sourced from the UK Met Office’s Operational Sea Surface Temperature and Ice Analysis (OSTIA) system (*50*, *51*), which provides daily gap-free foundation SST maps at a 0.05° horizontal resolution from October 1981 onward. The SSH data are derived from the Data Unification and Altimeter Combination System (DUACS) (*52*, *53*), delivering daily SLA at a 0.125° resolution from 1993 onward and a mean sea level (MSL) based on the 1993–2012 period. The SSH is computed by summing the SLA and MSL. In situ T/S profiles are obtained from the second version of the CODC Global Ocean Temperature and Salinity Profile Science Database (CODC-v2) (*54*), which supplies quality-controlled near-real-time observations. Before the fusion process, all satellite data are spatially interpolated onto the GLORYS reanalysis grid. Anomalies for SST, SSH (SSTA, SSHA), and in situ T/S profiles are computed relative to the climatological mean seasonal cycles during 1993 to 2022. It should be noted that some observations such as the gridded SLA are updated after their first release. Therefore, the observations used by WenHai-E2E may have higher quality than their counterparts by GLO12v4, which could partially contribute to the improved accuracy of initial condition of WenHai-E2E than GLO12v4.

The forecast performance of WenHai-E2E and GLO12v4 is evaluated against observational datasets following the GODAE Ocean-View IV-TT Class 4 (*16*), including the T/S profiles obtained from the CODC-v2, along-track Level-3 SLA from DUACS, and SST and 15-m horizontal current from drifter observations provided by the Global Drifter Program (*55*). Hourly drifter records are bin-averaged to compute the daily mean values.

### GLORYS reanalysis

The Copernicus Global 1/12° Oceanic and Sea Ice GLORYS12 Reanalysis (*56*) (GLORYS reanalysis for short) is developed by Mercator Océan. GLORYS reanalysis provides a state-of-the-art representation of the ocean and sea ice variabilities from daily to decadal scales over the altimetry period. The numerical model components are based on the NEMO platform (*57*). It has a grid with 1/12° horizontal resolution and 50 vertical levels. The atmospheric forcings are from the ERA-Interim atmospheric reanalysis (*58*) produced by the ECMWF. It uses the reduced-order Kalman filter to conduct DA every 7 days. Assimilated variables include the satellite-measured SLA, SST and sea ice concentration, and in situ T/S profiles.

### Atmospheric datasets

The atmospheric datasets for WenHai-E2E and GLO12v4 are obtained from ECMWF real-time forecast outputs from the IFS model. Variables fed into the bulk formulae module of WenHai-E2E comprise 10-m zonal and meridional winds, mean sea level pressure, 2-m air temperature and dewpoint temperature, surface net shortwave radiation, surface downward longwave radiation, and precipitation rate. Before air-sea flux calculations, all accumulated atmospheric fields are converted to daily mean fields. Despite both models being forced with atmospheric forecast datasets from the same operational system, GLO12v4 uses those with 0.1° spatial resolution, whereas WenHai-E2E only accesses the open data with a coarser 0.25° resolution.

### Fusion model

#### Procedure overview

A two-step framework is used to reconstruct three-dimensional gridded T/S fields from satellite and in situ observations. First, a mapping model is applied to generate a background field (first guess) of temperature and salinity anomalies relative to the climatological seasonal cycle based on satellite-derived SSTA and SSHA. The climatological seasonal cycle is computed on the basis of the GLORYS reanalysis during 1993–2022. Subsequently, this background field is refined on the basis of in situ profiles of T/S anomalies. Last, the anomalous fields of temperature and salinity are added back on their climatology to yield the temperature and salinity.

#### Mapping processes

Mapping the three-dimensional anomalous T/S fields from SSTA and SSHA is achieved through a geographically and seasonally varying coefficient (GSVC) linear regression model (*27*). The GSVC model accounts for geographical and seasonal variations in the surface-subsurface relationship arising from changes in the Coriolis parameter and ocean stratification. A key practical advantage of the GSVC model is its applicability for operational forecasting, as the regression coefficients, once trained on historical reanalysis data, are assumed to be temporally invariant across years.

Assume the ocean state (i.e., temperature or salinity) anomaly y(s,t) as a spatio-temporal Gaussian random process, with s denoting the spatial coordinate and t the time coordinate. The values of y(s,t) can be mapped from SSTA and SSHA via the GSVC model

$$y\left(s,t\right)=\beta_{1}\left(s,t\right)\cdot \text{SSTA}\left(s,t\right)+\beta_{2}\left(s,t\right)\cdot \text{SSHA}\left(s,t\right)+\eta \left(s,t\right)$$ (1)

where β is the geographically and seasonally varying regression coefficient estimated based on the GLORYS reanalysis dataset during 1993–2018 and η is the residue term representing subsurface ocean state anomaly not predictable from SSTA and SSHA. In the GSVC model, the varying coefficient β at each location and calendar day is estimated via locally weighted least squares using a spatio-temporal Gaussian kernel with a zonal bandwidth of 2°, a meridional bandwidth of 1°, and a temporal bandwidth of 30 days.

#### Refining processes

Now, the spatial resolution of operational in situ T/S profiles is limited to ∼300 km. This is adequate to resolve large-scale but not mesoscale ocean processes. Accordingly, in situ T/S profiles are only capable to refine the large-scale background T/S fields derived from the GSVC model. Hereinafter, the large-scale motions are extracted using a 4° × 4° moving mean filter.

The refining process is formulated within a Bayesian framework as a maximum a posteriori (MAP) estimation problem. Let $y_{L}=y_{L}\left(s,t\right)$ denote the large-scale ocean state on a predefined grid $s={\left(s_{1},s_{2}\ldots s_{N}\right)}^{T}$ at time t, and let z represent a set of irregularly spaced in situ observations. The objective is to find

$$y_{L}^{\text{MAP}}=\underset{y_{L}}{\text{argmax}}p\left(y_{L}|\;z\right)$$ (2)

where p represents the probability density function. According to the Bayesian theorem

$$p\left(y_{L}|z\right)\propto p\left(z|y_{L}\right)p\left(y_{L}\right)$$ (3)

The solution of $y_{L}^{\text{MAP}}$ thus requires explicitly modeling the prior $p\left(y_{L}\right)$ and the likelihood $p\left(z|y_{L}\right)$, respectively.

The large-scale ocean state $y_{L}$ is assumed to be a spatial Gaussian process

$$y_{L}∼\text{Gau}\left({\hat{y}}_{L},\Sigma_{\mathrm{yy}}\right)$$ (4)

where Gau represents the Gaussian distribution, ${\hat{y}}_{L}$ is the large-scale background field obtained from the GSVC model, and $\Sigma_{\mathrm{yy}}$ is the covariance matrix estimated as the sample covariance of the large-scale residuals $\eta_{L}=y_{L}-{\hat{y}}_{L}$ computed from the GLORYS reanalysis during 2019–2023.

The observation model relating in situ measurements z to the large-scale state $y_{L}$ is expressed as

$$z={\mathrm{Hy}}_{L}+\varepsilon$$ (5)

where H is an observation operator that spatially interpolates the gridded state $y_{L}$ onto the irregular in situ measurement locations and ε is a catch-all for the random errors including the representation errors and measurement errors. Assuming ε follows a spatial Gaussian process with zero mean and covariance matrix $\Sigma_{\varepsilon \varepsilon }$, the conditional distribution of z given $y_{L}$ is

$$z|y_{L}∼\text{Gau}\left({\mathrm{Hy}}_{L},\Sigma_{\varepsilon \varepsilon }\right)$$ (6)

Substituting Eqs. 4 to 6 into Eq. 3 gives rise to the $y_{L}^{\text{MAP}}$

$$y_{L}^{\text{MAP}}={\hat{y}}_{L}+\Sigma_{\text{yy}}H^{T}{\left({\mathrm{H\Sigma}}_{\text{yy}}H^{T}+\Sigma_{\varepsilon \varepsilon }\right)}^{-1}\left(z-H{\hat{y}}_{L}\right)$$ (7)

The measurement errors are due to the manufacturer-quoted sensor inaccuracies. They are much smaller in magnitude compared to representation errors described below and can thus be omitted. The representation errors arise from two primary sources. First, as the focus is on estimating $y_{L}$, variations of y contributed by mesoscale and submesoscale motions are representation errors. The mesoscale motions are the dominant contributor (*59*), and their contribution can be estimated based on the GLORYS reanalysis. Second, in view that the ocean state anomalies at different times are correlated due to the long memory of the ocean, in situ T/S profiles within a 1-month window preceding time t are used for estimating $y_{L}$. This introduces an additional representation error arising from changes in $y_{L}$ over time, which can be again estimated on the basis of the GLORYS reanalysis. The 1-month window is selected as most of the ocean has a decorrelation time scale longer than 1 month (fig. S7). Sensitivity tests suggest that it is slightly superior over a 15-day window and a 60-day window in terms of the overall forecast performance of WenHai-E2E (fig. S8). This may be because a 15-day window is too short to fully exploit useful observations, whereas a 60-day window is overly long compared to the decorrelation time scales in energetic regions such as the western boundary currents and Antarctic circumpolar current (fig. S7). We note that a single window length of 1 month is unlikely to be optimal everywhere, as the decorrelation time scale varies substantially with space (fig. S7). It is possible that the quality of initial condition can be further improved by using a spatially varying window length and carefully tuning its values.

#### Reconstruction performance

To evaluate the effects of mapping and refining processes on the quality of reconstructed initial condition, we validate the climatological seasonal cycle, the prior estimate mapped from the GSVC model, and the posterior estimate from the Bayesian model against in situ APB temperature profiles that are not exploited by the fusion model. The prior estimate reduces the reconstruction error relative to the climatological seasonal cycle, while the posterior estimate further improves the reconstruction after incorporating in situ T/S profiles (fig. S9). This confirms the complementary roles of satellite and in situ observations in constructing three-dimensional T/S fields.

#### Sea-ice mask

To ensure the reliability of satellite-derived sea surface signals, regions with sea-ice coverage are excluded. On the basis of the OSTIA sea ice concentration product, a grid cell at some time is defined as “ice-covered” when its concentration exceeded 30%. Grid cells where the fraction of “ice-covered” days exceeds 50% are masked and excluded from the analysis. The remaining area is shown in fsig. S5. The criterion for defining the sea-ice mask is somewhat arbitrary. Nevertheless, the forecast performance globally is insensitive to the chosen thresholds.

### Forecast model

#### Training datasets

The forecast model is trained on the GLORYS reanalysis during 1993–2018, satellite observations and constructed three-dimensional T/S from the fusion model during 2019–2023 and ERA5 reanalysis (*60*) during 1993–2023.

#### Input and output details

The forecast model is designed to forecast the tendency of daily mean ocean variables led by 1 to 10 days $\left\{∆O_{t}^{l}=O_{t}^{l}-O_{t}^{l-1},l\in \left[1,N\right]\right\}$ in an autoregressive way, given the initial condition $O_{t}^{0}$ and the surface atmospheric variables $\left\{A_{t}^{l},l\in \left[0,N-1\right]\right\}$, where t is an arbitrary date index and l is the forecast lead time. The bold characters indicate that $O_{t}^{l}$ and $A_{t}^{l}$ consist of multiple variables at multiple grid points. In specific, $O_{t}^{l}$ contains three-dimensional T/S and SSH. The forecast model shares the same first 40 depth levels from the GLORYS reanalysis, where the maximum depth is 1942 m (fig. S10). The $O_{t}^{l}$ denotes the three-dimensional T/S and SSH $O_{t}^{l}=\left\{T_{t}^{l},S_{t}^{l},{\text{SSH}}_{t}^{l}\right\}$ and shares the same horizontal grid points as the GLORYS reanalysis. Accordingly, $T_{t}^{l}$ and $S_{t}^{l}$ have a dimension size of $N_{D}\times N_{\text{lat}}\times N_{\text{lon}}$, ${\text{SSH}}_{t}^{l}$ has a dimension size of $1\times N_{\text{lat}}\times N_{\text{lon}}$, with $N_{D}=40$, $N_{\text{lat}}=2041$, and $N_{\text{lon}}=4320$. The value of $N_{D}$ can be seen as the channel dimension in the computer vision domain.

The $A_{t}^{l}$ contains 10-m zonal wind, 10-m meridional wind, mean sea level pressure, 2-m temperature and 2-m dewpoint temperature. We implement a bulk formule module to compute the air-sea fluxes based on the surface values of $O_{t}^{l}$ and $A_{t}^{l}$ to better represent the atmospheric forcings in the forecast model. Specifically, $A_{t}^{l}$ are first horizontally remapped to the oceanic grids, and then the bulk formula module computes the air-sea fluxes, including the surface latent heat flux, surface sensible heat flux, surface upward long-wave radiation flux, zonal wind stress, meridional wind stress, and evaporation rate. In addition, surface net short-wave radiation, surface downward long-wave radiation, and precipitation rate are obtained directly from the numerical weather forecasts. The surface net long-wave radiation flux is then derived by combining this downward component with the upward long-wave radiation. Hereinafter, $F_{t}^{l}$ is used to refer to the eight air-sea flux variables, whose dimension size is $N_{F}\times N_{\text{lat}}\times N_{\text{lon}}$ with $N_{F}=8$. The $F_{t}^{l}$ and $O_{t}^{l}$ are fed into the forecast model to forecast $∆O_{t}^{l}$. Once $∆O_{t}^{l}$ is derived, it is added back on $O_{t}^{l}$ to obtain $O_{t}^{l+1}$. Then, $F_{t}^{l+1}$ is obtained on the basis of the surface values of $O_{t}^{l+1}$ and $A_{t}^{l+1}$. The above processes are repeated to make a sequence of forecasts autoregressively.

#### Architecture overview

The values of $O_{t}^{l}$ and $F_{t}^{l}$ are first normalized to range from 0 to 1 for each channel. The normalized variables are then processed by two independent preprocessing modules to reduce dimensionality. The outputs of pre-processing modules in a dimension of 287×574×H are then reshaped to 164738×H and added together before going through 10 homogeneous Swintransformer layers whose hidden dimension size H is 768 and window size is 7. Last, a post-processing module is implemented to restore the outputs of Swintransformer layers to the original dimension of $O_{t}^{l}$.

#### Pre- and postprocessing

Each variable in $O_{t}^{l}$ is first preprocessed by a horizontal padding block and a patch embedding block. Then, a feature aggregation block is implemented to concatenate and fuse outputs for different variables, followed by two down-sampling blocks. In the horizontal padding block, the periodic padding is applied in the longitudinal direction and the symmetric padding is applied in the latitudinal direction to properly represent the boundary conditions in the rectangular grid. We use 127, 128, 136, and 136 as the padding size for northern, southern, eastern, and western boundaries, respectively, resulting in a padded horizontal grid size of 2296×4592. The padding size is specifically designed so that the padded horizontal grid size is divisible by both the window size 7 and the down-sampling ratio 8 to avoid indivisibility during computations. Normalized and padded $O_{t}^{l}$ and $F_{t}^{l}$ are fed into the patch embedding blocks, which are convolution layers whose kernel size and stride (also known as the patch size) is chosen as 2 in this study. The outputs of the patch embedding blocks for $T_{t}^{l}$, $S_{t}^{l}$, and ${\text{SSH}}_{t}^{l}$ are with the dimension of 192×1148×2296, 192×1148×2296, and 32×1148×2296, respectively. We choose a relatively small patch size of 2 to make the forecast model better preserve the mesoscale variabilities. The feature aggregation block first concatenates these outputs to a tensor with the dimension of (192+192+32)×1148×2296, which is then projected to a tensor with the dimension of H×1148×2296 by a convolution layer with kernel size 1 and stride 1. Both the two down-sampling blocks use convolution layers with kernel size 3 and stride 2 to further reduce the data dimension to H×574×1148 and H×287×574 sequentially, followed by permuting the dimension to 287×574×H. The post-processing module consists of two up-sampling blocks, a feature decomposition block, three linear layers, and a pad-back block. The up-sampling blocks are two-dimensional transposed convolution layers with kernel size 2 and stride 2. The first up-sampling block changes the dimension of reshaped output from the Swin transformer layers, concatenated with the second down-sampling block output along the hidden dimension, from 2H×287×574 to H×574×1148. The second up-sampling block changes the first up-sampling block output, concatenated with the first down-sampling block output along the hidden dimension, from 2H×574×1148 to H×1148×2296. The data are then concatenated with the patch embedding block output, followed by permuting the dimension to 1148×2296×2H. The decomposition block first projects the data dimension from 1148×2296×2H to 1148×2296×(192+192+32), which is then split into three outputs with the dimension of 1148×2296×192, 1148×2296×192 and 1148×2296×32. The three linear layers transform the output dimension to 1148×2296×160, 1148×2296×160 and 1148×2296×4, respectively. Then, the outputs are rearranged to the dimension of 40×2296×4592, 40×2296×4592, and 1×2296×4592, corresponding to $T_{t}^{l}$, $S_{t}^{l}$, and ${\text{SSH}}_{t}^{l}$ outputs. Last, the pad-back module removes the padded margins and restores the outputs to the original horizontal dimension of $O_{t}^{l}$.

#### Window-based Transformer

After being processed by the preprocessing module, inputs are permuted to numerous patch tokens with a sequence length of 164,738 to be fed into window-base attention blocks. Divided into 3362 windows, each window calculates self-attention with 49 tokens.

#### Land-sea ice mask

Throughout all processing steps, values on land and in ice-covered region are constantly set to 0. The land-sea ice mask used here is identical to that used in the fusion model.

#### Three-stage training strategy

The training process is divided into three stages. In the first stage, a base model is pretrained by minimizing a weighted mean absolute error (MAE) for $∆O_{t}^{1}$ integrated over the global ocean, i.e.

$$\text{min}∭{\left\|w\cdot \left(∆{\hat{O}}_{t}^{1}-∆O_{t}^{1}\right)\right\|}_{1}\mathrm{dV}\approx \text{min}\sum_{i}{\left\|w\left(i\right)\cdot \left(∆{\hat{O}}_{t,i}^{1}-∆O_{t,i}^{1}\right)\right\|}_{1}\delta V\left(i\right)$$ (8)

where $∆{\hat{O}}_{t,i}^{1}$ denotes the forecast tendency at the *i*-th grid cell, $∆O_{t,i}^{1}$ is the ground truth from the GLORYS reanalysis, δV(i) is the volume of the *i*-th ocean grid cell, and w(i) is a weight vector for different ocean variables whose value is chosen following (*12*).

During the second training stage, the pretrained model is finetuned on the GLORYS reanalysis to improve its performance at longer forecast lead times. This is done by optimizing the accumulated MAE over a sequence of autoregressive forecasts, i.e.

$$\text{min}\sum_{l=1}^{M}\sum_{i}{\left\|w\left(i\right)\cdot \left(∆{\hat{O}}_{t,i}^{l}-∆O_{t,i}^{l}\right)\right\|}_{1}\delta V\left(i\right)$$ (9)

where $O_{t,i}^{l}$ is obtained from GLORYS reanalysis, and we set M=5.

During the third training stage, the fine tuned model based on the GLORYS reanalysis is further fine tuned on the basis of the satellite observations and constructed three-dimensional T/S from the fusion model during 2019–2023 to make the forecast model adopt to the deployment scenario, i.e.,

$$\text{min}\sum_{l=1}^{M}\sum_{i}{\left\|w\left(i\right)\cdot \left(∆{\hat{O}}_{t,i}^{l}-∆O_{t,i}^{l}\right)\right\|}_{1}\delta V\left(i\right)$$ (10)

where $O_{t,i}^{l}$ is obtained from the fusion model. The third training stage improves the forecast skills of WenHai-E2E for all the ocean variables (fig. S11).

### Retrieval model

The retrieval model retrieves the three-dimensional horizontal currents based on the SSH, three-dimensional T/S, and air-sea fluxes. It shares similar neural network configurations as the forecast model. Only their differences are elaborated here.

#### Input and output details

The retrieval model is aimed to retrieve daily mean three-dimensional horizontal currents in the upper ocean $\left\{C_{t}^{l},l\in \left[0,N\right]\right\}$, given the SSH and three-dimensional T/S $O_{t}^{l}$ and air-sea flux variables $F_{t}^{l}$ at the same time. $C_{t}^{l}$ contains zonal and meridional currents $C_{t}^{l}=\left\{u_{t}^{l},v_{t}^{l}\right\}$. The retrieval model shares the same model grid with the forecast model. $u_{t}^{l}$ and $v_{t}^{l}$ have a dimension of $N_{D}\times N_{\text{lat}}\times N_{\text{lon}}$ with $N_{D}=40$, $N_{\text{lat}}=2041$, and $N_{\text{lon}}=4320$.

#### Model architecture

The architecture of the retrieval model is the same as that of the forecast model, except that the target feature size is changed to (192+192) in the feature decomposition block, and accordingly there are two linear layers in post-processing module. The linear layer changes the output from the previous step from 1148×2296×192 to 1148×2296×160. The data are then rearranged to have a dimension of 40×2296×4592. Last, the retrieval model outputs for $u_{t}^{l}$ and $v_{t}^{l}$ are restored to the original horizontal dimension of $C_{t}^{l}$ by the pad-back module.

#### Training strategy

The training process is conducted by optimizing a weighted MAE for $C_{t}^{0}$ integrated over the global ocean, i.e.

$$\text{min}∭{\left\|{\hat{C}}_{t}^{0}-C_{t}^{0}\right\|}_{1}\mathrm{dV}\approx \text{min}\sum_{i}{\left\|{\hat{C}}_{t,i}^{0}-C_{t,i}^{0}\right\|}_{1}\delta V\left(i\right)$$ (11)

where ${\hat{C}}_{t,i}^{0}$ represents the retrieved currents at the *i*-th grid cell, and the ground truth $C_{t,i}^{0}$ is obtained from the GLORYS reanalysis.

### Verification metrics

The verification metrics are computed generally following (*12*) and briefly described here. The RMSE is defined as

$$\text{RMSE}=\sqrt{\frac{\sum_{i=1}^{N}\varepsilon_{i}^{2}}{N}}$$ (12)

where $\varepsilon_{i}$ is the difference of the match-up between the observations and forecasts interpolated to sites of observations and N is the total number of match-ups.

The PSS is defined as

$$\text{PSS}=1-\frac{\text{RMSE}}{{\text{RMSE}}_{p}}$$ (13)

where ${\text{RMSE}}_{p}$ is the RMSE of a persistent forecast that uses the 1-day-lead forecast as the forecast at longer forecast lead times.

The CRPS evaluated against some observation $V_{i}$ is defined as

$$\text{CRPS}=\int_{-\infty }^{+\infty }{\left[F\left(x\right)-H\left(x\geq V_{i}\right)\right]}^{2}\mathrm{dx}$$ (14)

where F(x) is the cumulative distribution function of pseudo ensemble forecast and *H* is the Heaviside function. The pseudo ensemble forecast is formed by collecting forecast values within a 1° × 1° box centered on the grid cell closest to the site of $V_{i}$. All three metrics are first computed for each time, and then their median values among different times are adopted following the protocol of the GODAE Ocean-View IV-TT Class 4.

### Computation of geostrophic currents

Geostrophic currents are computed as

$$u_{g}=-\frac{1}{f\rho_{0}}\frac{\partial p}{\partial y}$$ (15)

$$v_{g}=\frac{1}{f\rho_{0}}\frac{\partial p}{\partial x}$$ (16)

where $u_{g}$ and $v_{g}$ are the geostrophic zonal and meridional currents, g is the gravitational acceleration, f is the Coriolis parameter, $\rho_{0}$ is the reference seawater density, and p is the pressure computed from the hydrostatic balance.

### Eddy identification

Eddy identification is conducted on the basis of the gridded SLA using an automated geometry-based algorithm (*41*, *61*). The forecast SSH from WenHai-E2E and GLO12v4 are first interpolated onto the grid of satellite-measured SLA and then converted to SLA by subtracting the MSL of the GLORYS reanalysis during 1993–2012. Closed contours of SLA are derived at an interval of 1 cm. Within each nest of contours, the geometric center of the innermost loop is designated as the eddy center. The eddy’s edge is defined by the outermost closed contour that contains this single center. The amplitude is calculated as the absolute SLA difference between the center and this edge. The equivalent radius is computed as the radius of a circle whose area matches the area enclosed by the edge. In view of deficiencies of the satellite-measured SLA in identifying mesoscale eddies with relatively small radius and amplitude, only eddies with amplitude > 5 cm and radius > 50 km are retained.

To evaluate the capacity of WenHai-E2E in forecasting eddy occurrence, eddies in observations and forecasts are matched and then a binary classification framework is adopted. A forecast eddy is considered to match some observed eddy if its center lies inside the boundary polygon of that observed eddy and the two eddies share the same polarity. An observed eddy matched by the forecast is counted as a true positive (TP), an unmatched observed eddy as a false negative (FN), and a forecast eddy unmatched by the observation as a false positive (FP). The hit rate, miss rate, and false alarm rate are defined as (*62*)

$$\text{Hit}\;\text{rate}=\frac{\text{TP}}{\text{TP}+\text{FN}}$$ (17)

$$\text{Miss rate}=\frac{\text{FN}}{\text{TP}+\text{FN}}$$ (18)

$$\text{False alarm rate}=\frac{\text{FP}}{\text{TP}+\text{FP}}$$ (19)
