# Supplementary material for: An end-to-end machine-learning system for global eddy-resolving ocean forecast
Source: Sci Adv. 2026 Nov 13;12(46):eaeh9058. doi: 10.1126/sciadv.aeh9058 (PMC13763865; doi:10.1126/sciadv.aeh9058)
Supplement: Supplementary file 1 [file sciadv.aeh9058_sm.pdf]

Science Advances
AAAS

Supplementary Materials for

An end-to-end machine-learning system for global eddy-resolving ocean forecast

Xiang Zhang *et al.*

Corresponding author: Zhao Jing, jingzhao@ouc.edu.cn; Lixin Wu, lxwu@ouc.edu.cn

Sci. Adv. **12**, eaeh9058 (2026)
DOI: 10.1126/sciadv.aeh9058

This PDF file includes:

Figs. S1 to S11

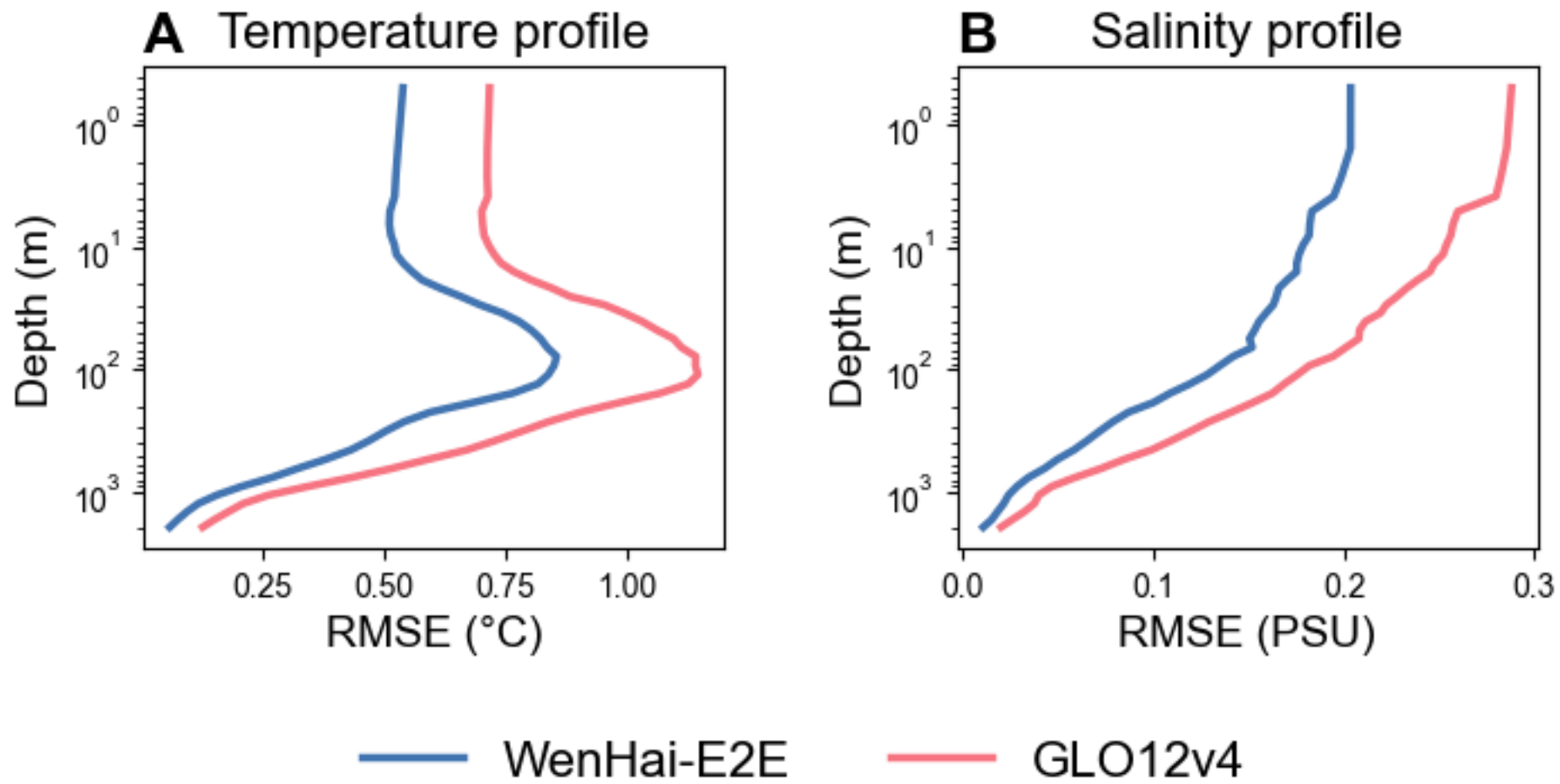

Fig. S1. Forecast root mean square error (RMSE) of temperature and salinity profiles of WenHai-E2E (blue) and GLO12v4 (red) as a function of depth. Globally averaged RMSE (the lower, the better) for the 10-day-lead forecast temperature profile (**A**) and salinity profile (**B**).

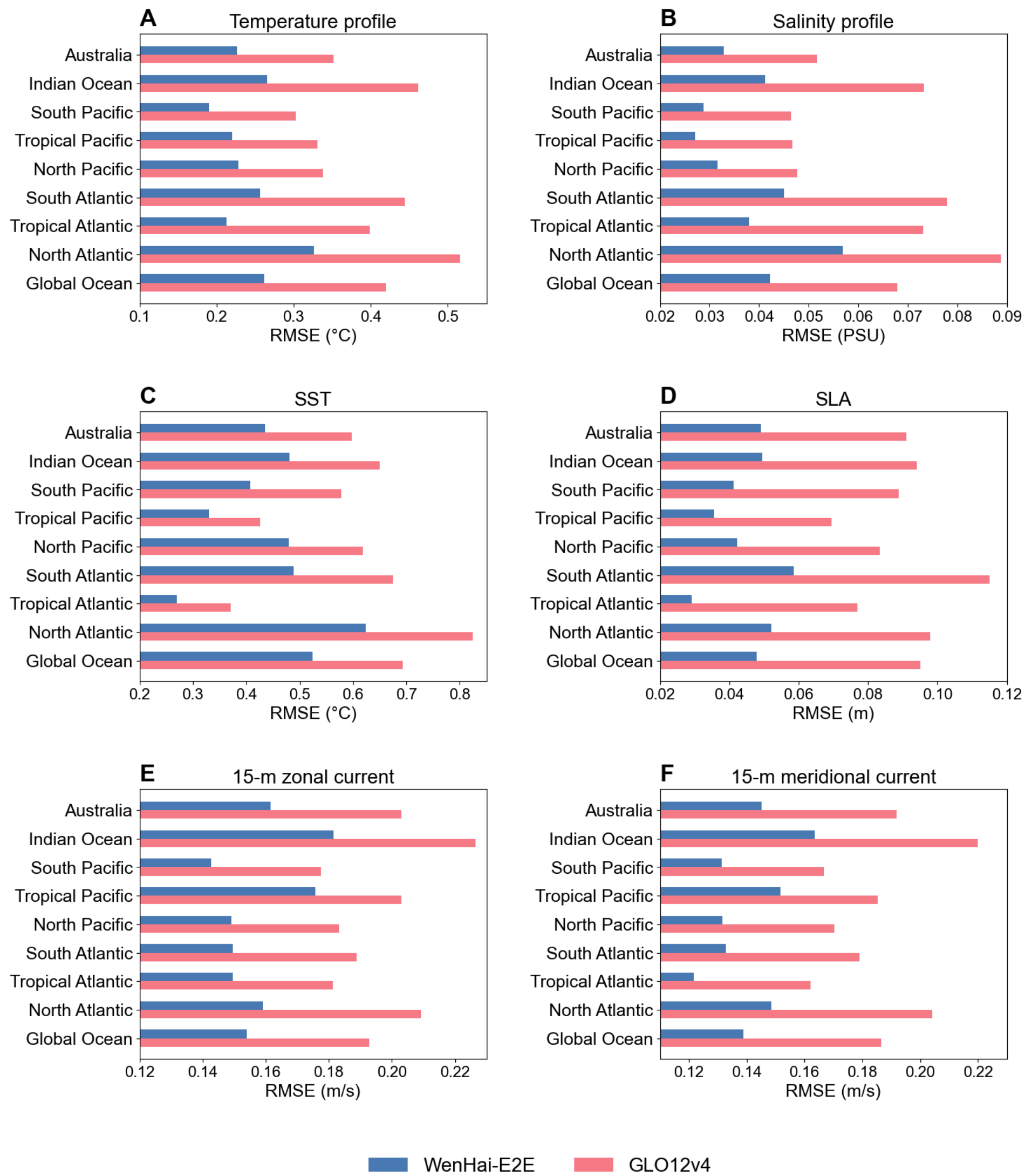

Name	Longitudes	Latitudes
Global Ocean	180W~180E	90S~90N
North Atlantic	100W~31E	0N~70N
Tropical Atlantic	70W~30E	20S~20N
South Atlantic	70W~30E	60S~0S
North Pacific	100E~77W	0N~65N
Tropical Pacific	90E~70W	20S~20N
South Pacific	100E~70W	60S~0S
Indian Ocean	20E~120E	40S~31N
Australia	90E~180E	70S~20N

Fig. S2. Regional forecast root mean square error (RMSE) between WenHai-E2E (blue) and GLO12v4 (red). Regionally averaged RMSE (the lower, the better) of the 10-day-lead forecast temperature profile (**A**), salinity profile (**B**), SST (**C**), SLA (**D**), 15-m zonal current (**E**) and 15-m meridional current (**F**). RMSE for the temperature and salinity profiles is vertically averaged over the upper 2000 m. The regions are defined in accordance with the GODAE Ocean-View IV-TT Class 4 framework, as detailed in the accompanying table.

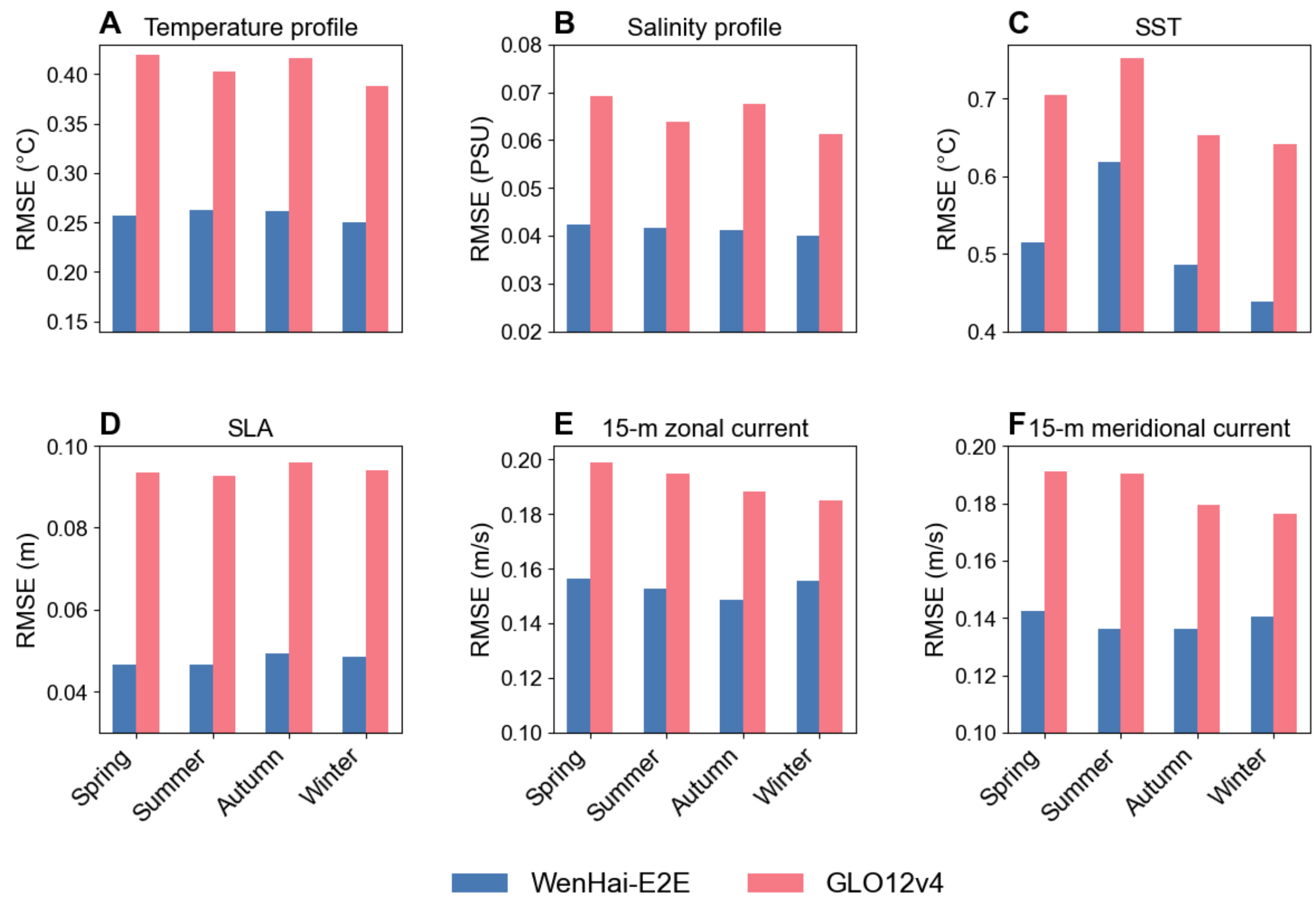

Fig. S3. Forecast root mean square error (RMSE) of the WenHai-E2E (blue) and GLO12v4 (red) in different seasons. A-F, Globally averaged RMSE for the 10-day-lead forecast of temperature profile (**A**), salinity profile (**B**), SST (**C**), SLA (**D**), 15-m zonal current (**E**) and 15-m meridional current (**F**) during spring, summer, autumn and winter. RMSE for temperature and salinity profile is vertically averaged over the upper 2000 m. Spring corresponds to MAM (SON), summer corresponds to JJA (DJF), autumn corresponds to SON (MAM), and winter corresponds to DJF (JJA) for the northern (southern) hemisphere.

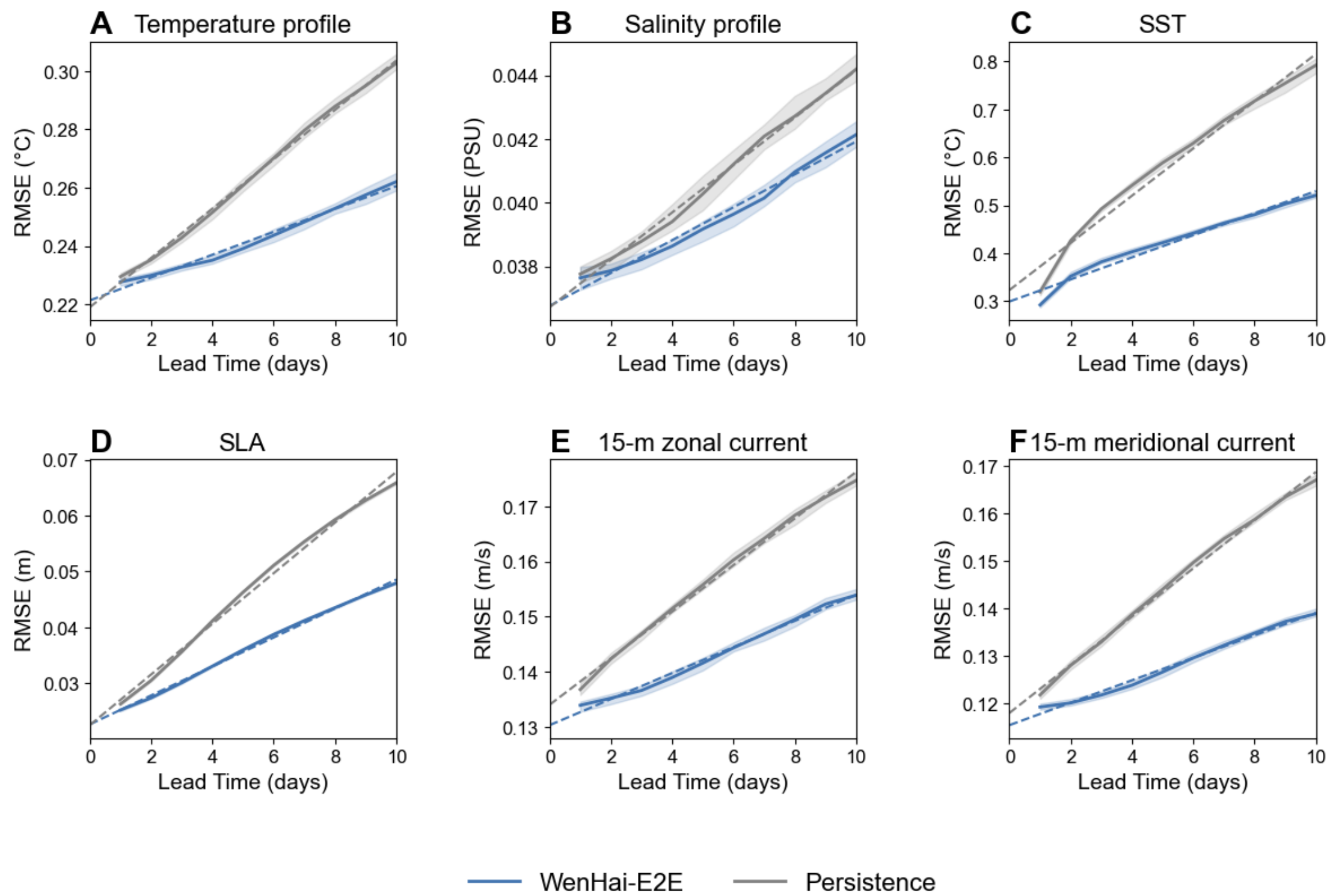

Fig. S4. Root mean square error (RMSE) of WenHai-E2E (blue) and persistence forecast (gray) at different forecast lead times. Forecast RMSE (the lower, the better) of the temperature profile (**A**), salinity profile (**B**), SST (**C**), SLA (**D**), 15-m zonal current (**E**) and 15-m meridional current (**F**) averaged globally as a function of forecast lead time. The zero-lead time represents the initial conditions whose RMSE is computed based on the linear fit (dashed line) of forecast RMSE. The persistence forecast keeps the WenHai-E2E initial condition unchanged at all forecast lead times. The RMSE for the temperature and salinity profile forecast is vertically averaged over the upper 2000 m. The shading corresponds to the 90% confidence interval estimated from a bootstrap method.

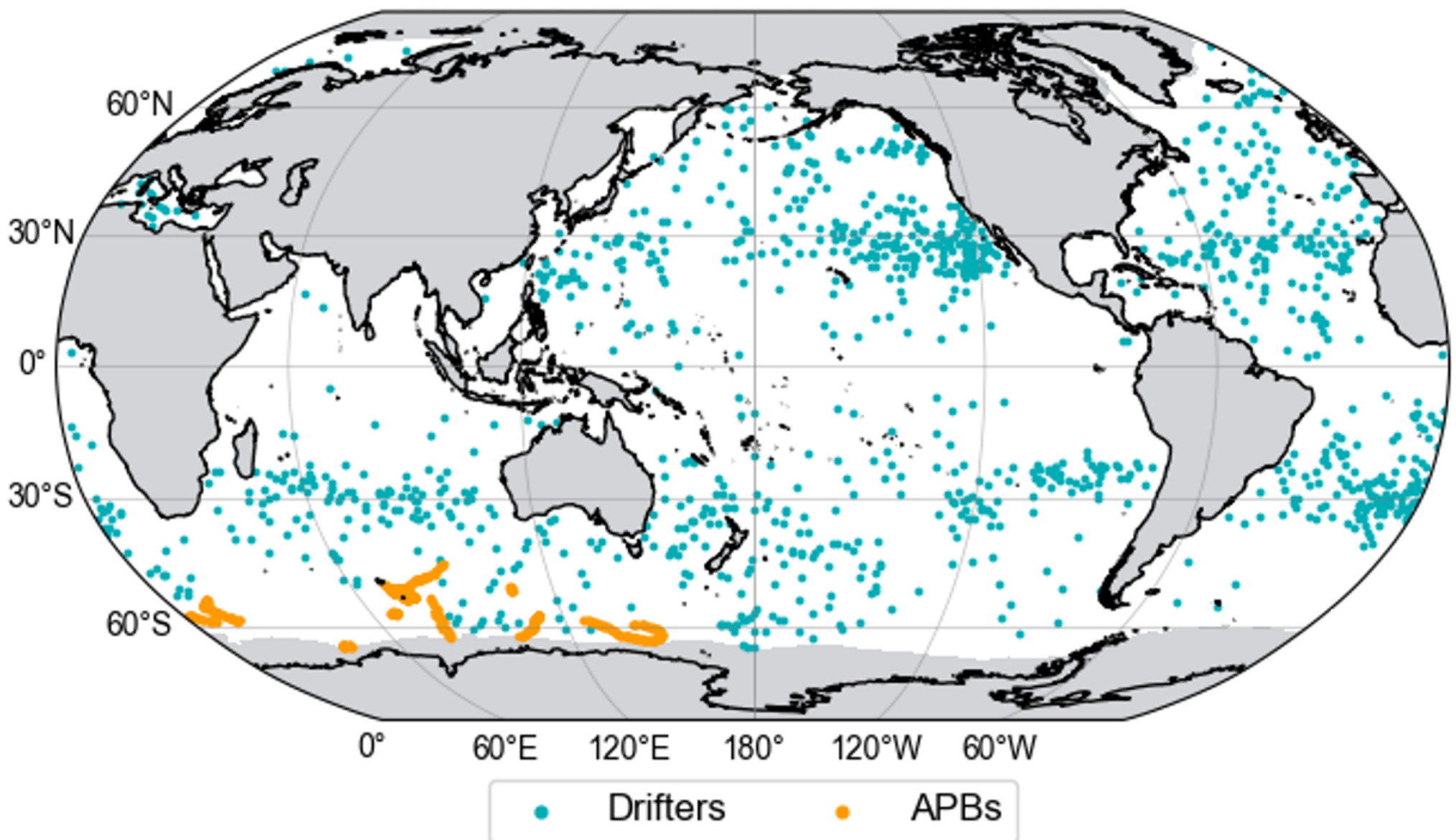

Fig. S5. Spatial distribution of observations. Blue dots denote surface drifters on a representative single day (9 April 2024), while yellow dots show all Autonomous Pinniped Bathythermograph (APB) profiles collected during the entire month of April 2024. The one-month APB composite illustrates its spatially sparse and uneven sampling compared to drifter. Grey regions indicate either land or areas covered by sea ice and excluded from the analysis.

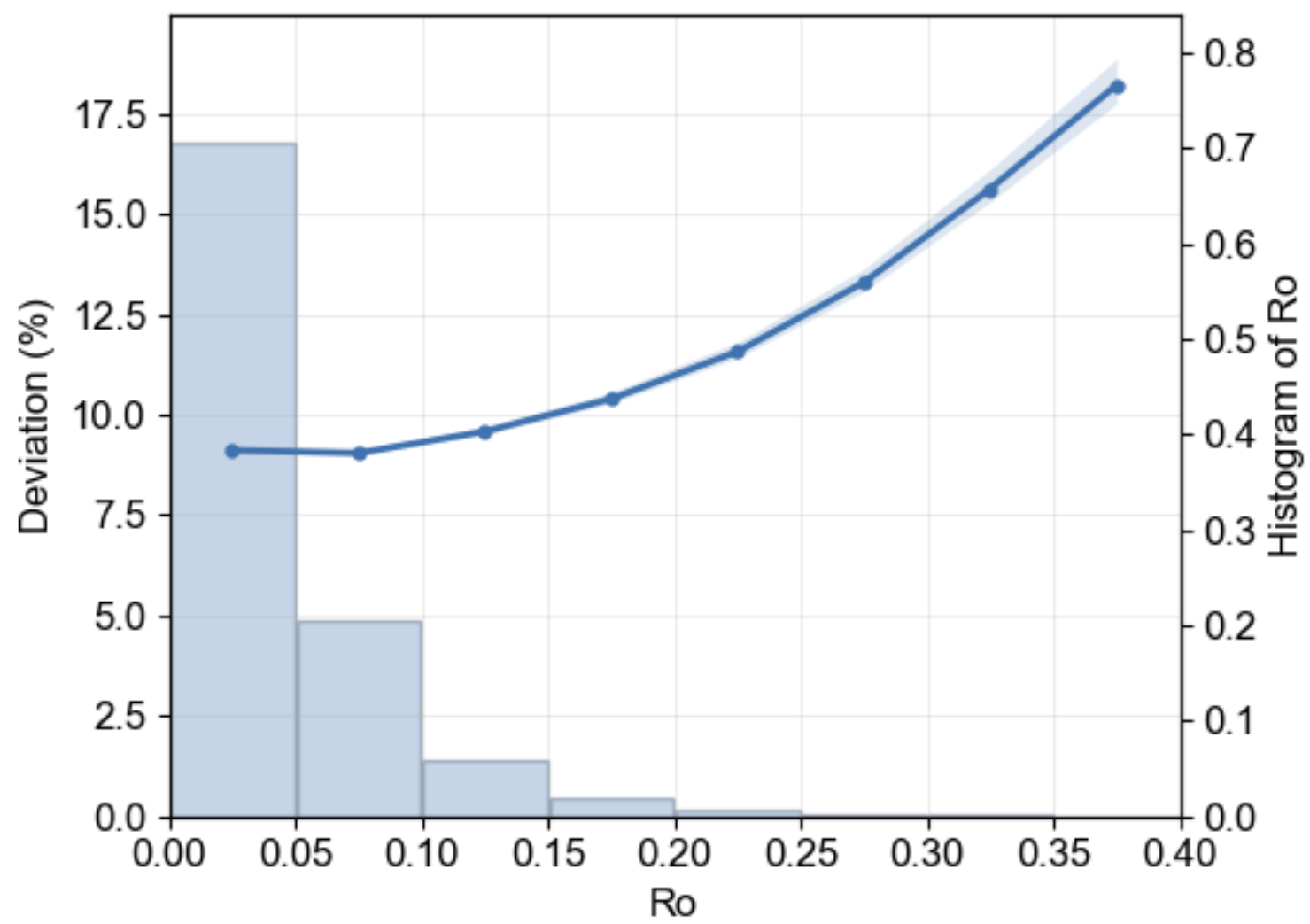

Fig. S6. Satisfaction of geostrophic balance under a small-Rossby number (Ro) regime for WenHai-E2E at the forecast lead time of 10 days. Deviation of horizonal currents from the geostrophic component as a function of Ro. Here the deviation is defined as the root-mean-square difference between the horizontal current and geostrophic component normalized by the root-mean-square geostrophic current. The root mean square is calculated over samples collected within 10°-60° for each bin of Ro. The shading corresponds to the 90% confidence interval estimated from a bootstrap method. The bars show the histogram of Ro with the bar width corresponding to the bin length.

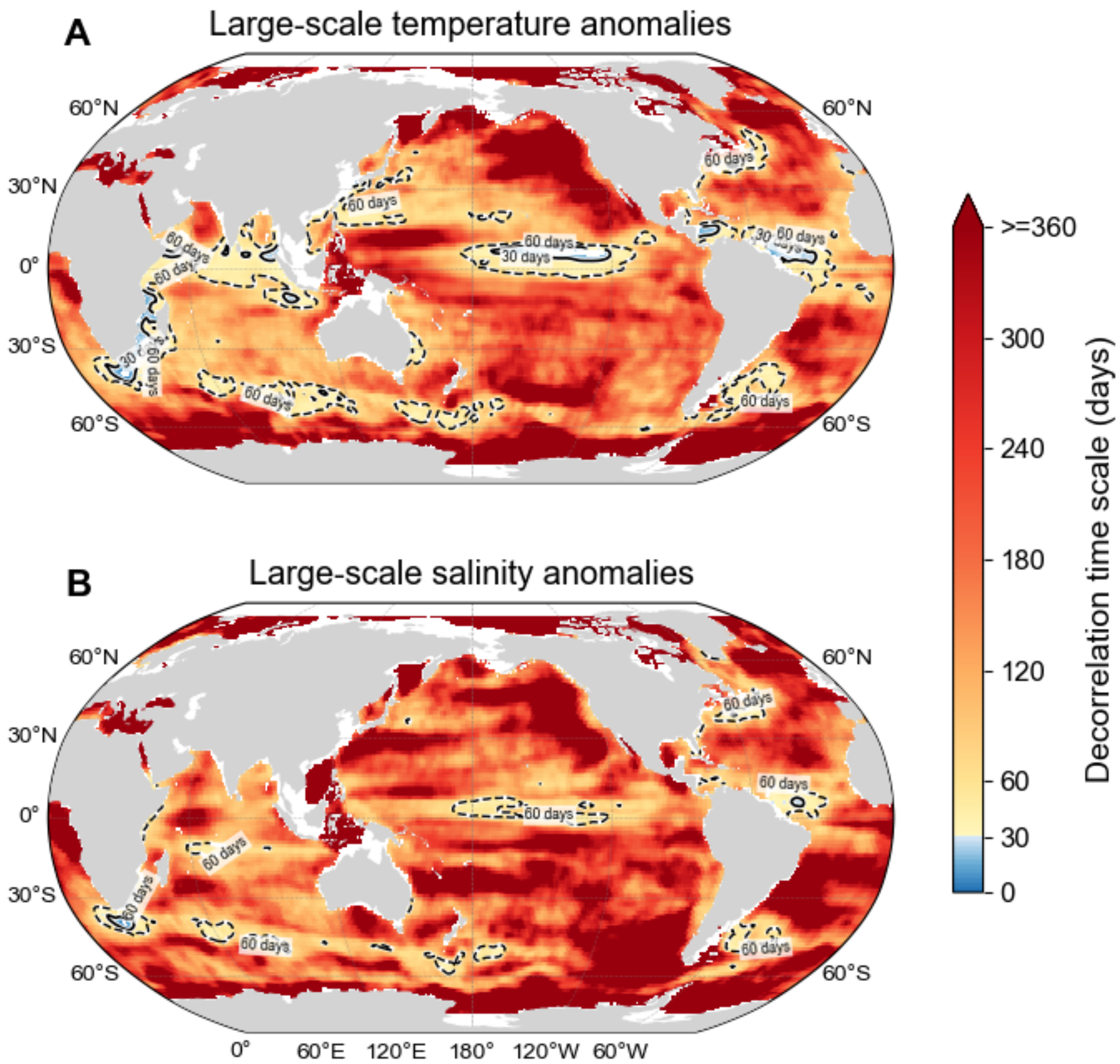

Fig. S7. Spatial distribution of the decorrelation time scale of large-scale temperature anomalies (A) and salinity anomalies (B) at 155.9 m depth. The decorrelation time scale is estimated from the GLORYS reanalysis during 1993-2020 as the e-folding time scale obtained by fitting an exponential decay to the autocorrelation over lags of 1-30 days. The solid and dashed black contours indicate the 30-day and 60-day thresholds, respectively.

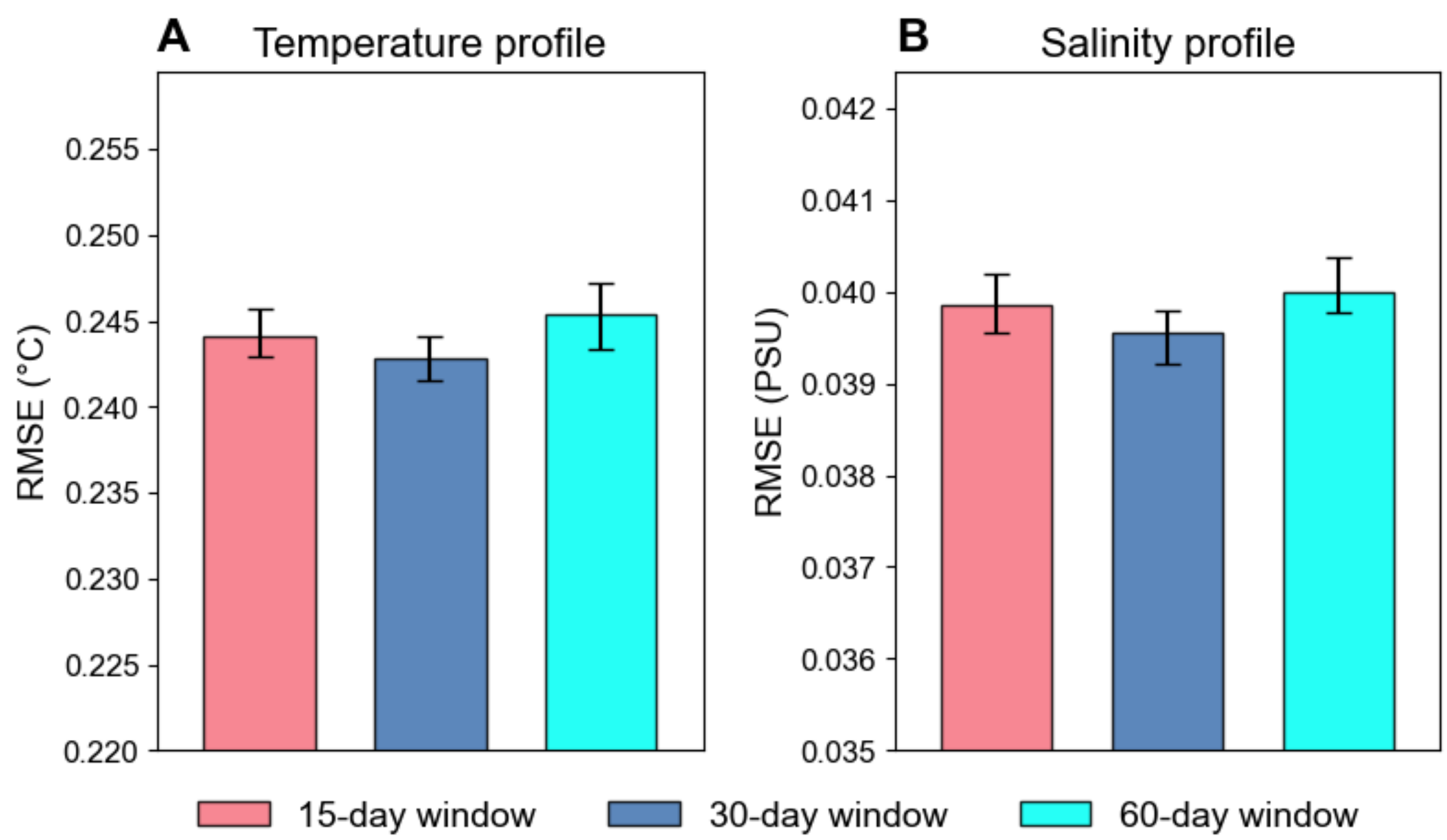

Fig. S8. Sensitivity of forecast performance to the window length used in the Fusion model. Global mean forecast RMSE (the lower, the better) of the temperature profile (**A**) and salinity profile (**B**) averaged during the forecast lead times from 1 day to 10 days over the upper 2000m. The red, blue, and cyan bars correspond to the results from the window length of 15, 30 and 60 days, respectively. The errorbar corresponds to the 90% confidence interval estimated from a bootstrap method.

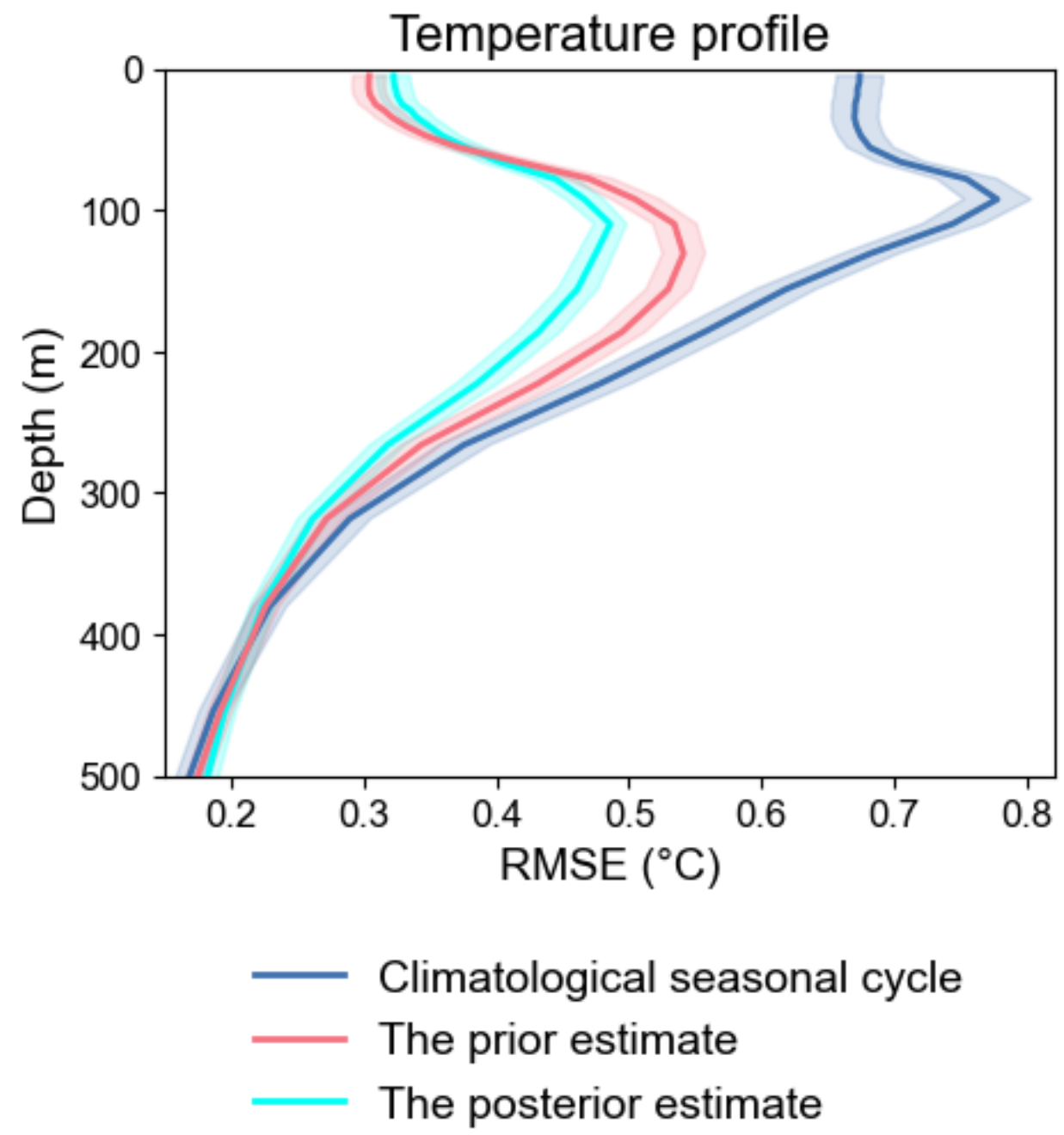

Fig. S9. Effects of mapping and refining processes of the fusion model on the quality of reconstructed initial condition. RMSE of climatological seasonal cycle (blue), the prior estimate mapped from the GSVC model (red), and the posterior estimate from the Bayesian model (cyan) validated against in situ APB temperature profiles. The shading corresponds to the 90% confidence interval estimated from a bootstrap method.

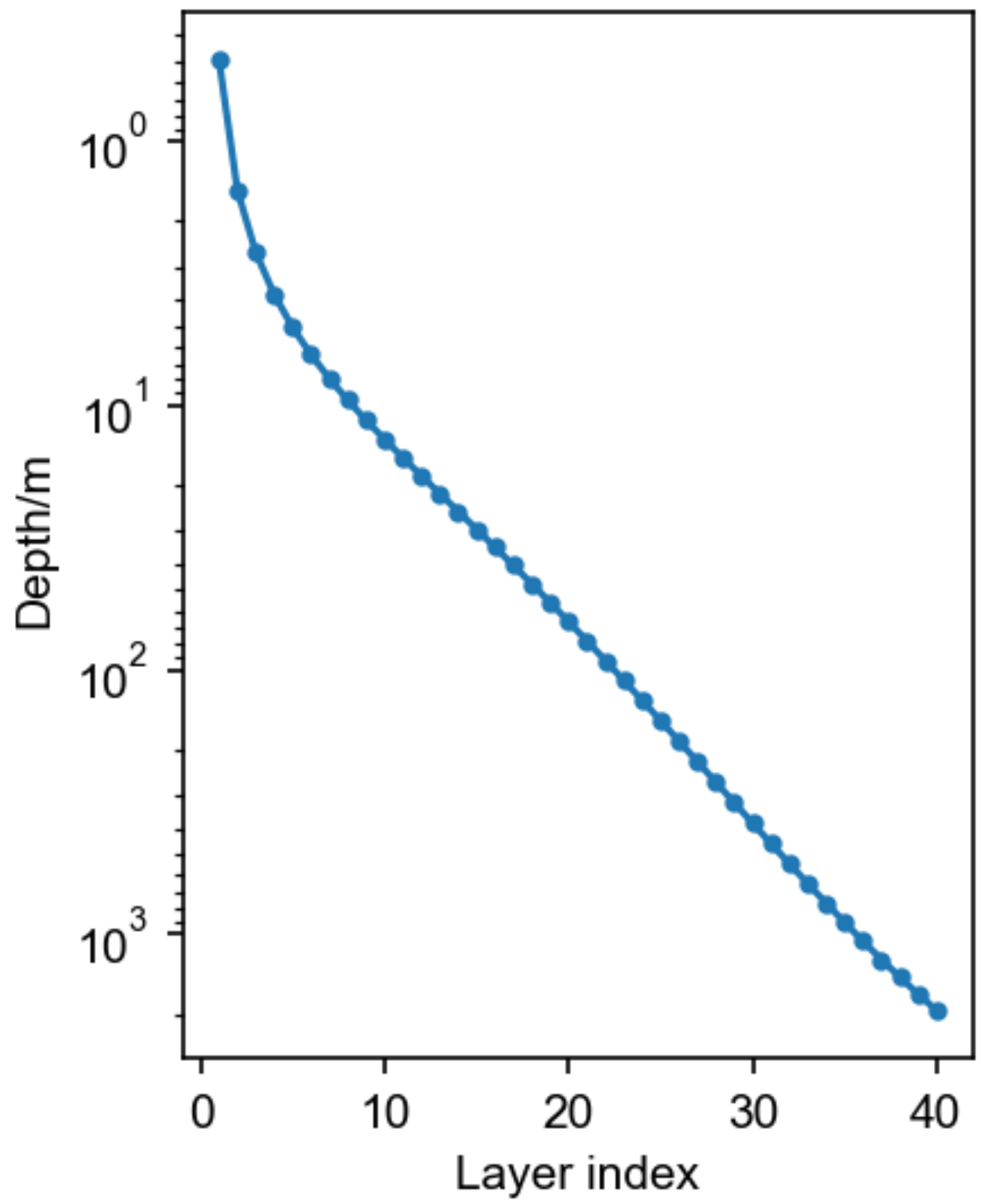

Fig. S10. Depth levels of WenHai-E2E.

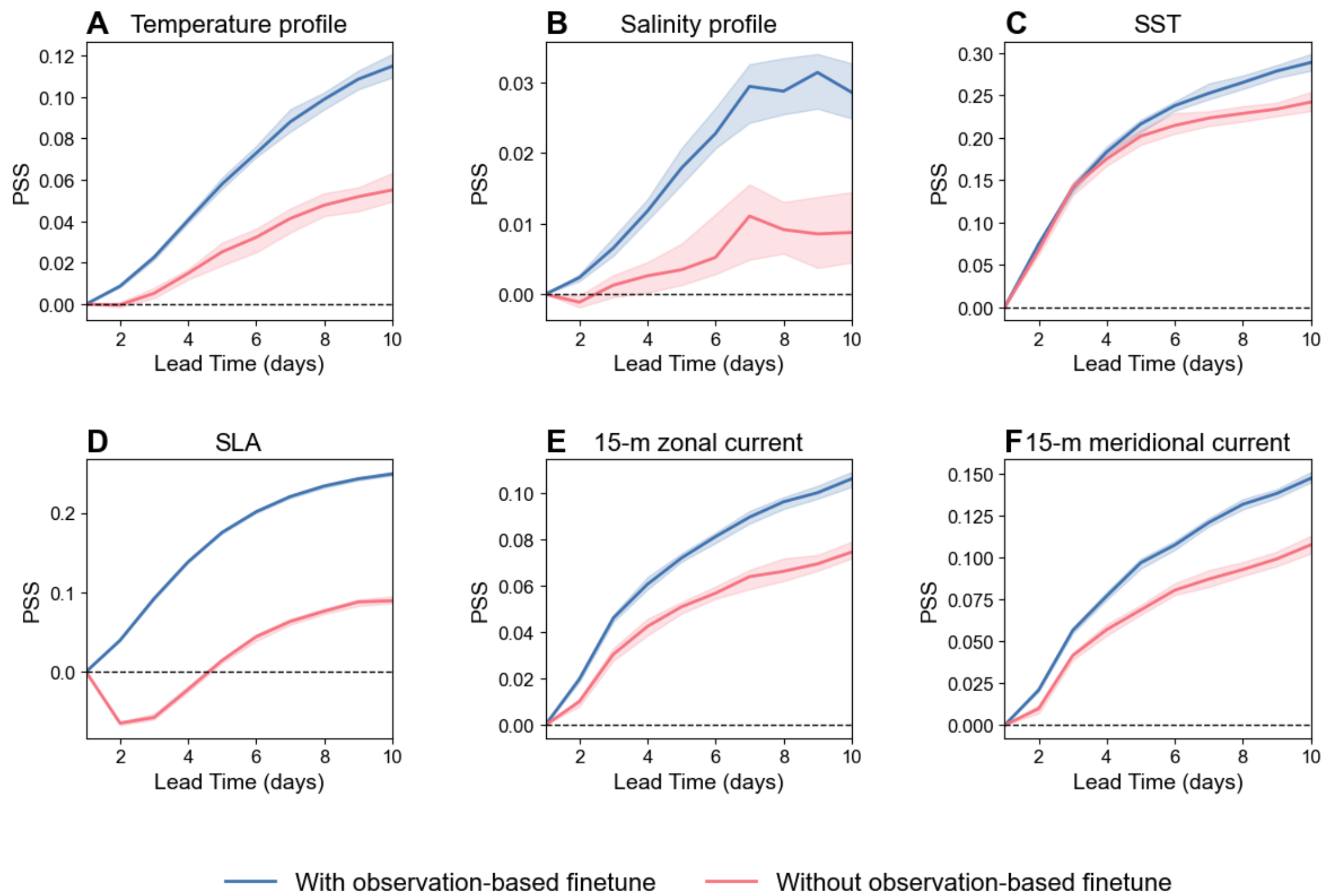

Fig. S11. Improved skill of the forecast model of WenHai-E2E via finetuning based on satellite observations and constructed three-dimensional temperature and salinity. Persistence forecast skill (PSS) of WenHai-E2E with (blue) and without (red) observation-based finetune. **A-F,** correspond to the PSS of the temperature profile, salinity profile, SST, SLA, 15-m zonal current and 15-m meridional current averaged globally as a function of forecast lead time. For the temperature and salinity profile forecast, the PSS is computed based on the vertically averaged RMSE over the upper 2000 m. The shading corresponds to the 90% confidence interval estimated from a bootstrap method.
